# Genetic and QTL analyses of sugar and acid content in sweet cherry (*Prunus avium* L.*)*

**DOI:** 10.1093/hr/uhae310

**Published:** 2024-11-06

**Authors:** Clara Gracia, Alejandro Calle, Ksenija Gasic, Esther Arias, Ana Wünsch

**Affiliations:** Departamento de Ciencia Vegetal, Centro de Investigación y Tecnología Agroalimentaria de Aragón (CITA), Avda. Montañana 930, Zaragoza 50059, Spain; Departamento de Producción Animal y Ciencia de los Alimentos, Instituto Agroalimentario de Aragón-IA2 (CITA-Universidad de Zaragoza), C. Miguel Servet 177, Zaragoza 50013, Spain; Departamento de Ciencia Vegetal, Centro de Investigación y Tecnología Agroalimentaria de Aragón (CITA), Avda. Montañana 930, Zaragoza 50059, Spain; Departamento de Producción Animal y Ciencia de los Alimentos, Instituto Agroalimentario de Aragón-IA2 (CITA-Universidad de Zaragoza), C. Miguel Servet 177, Zaragoza 50013, Spain; Fruit Production, IRTA, Fruitcentre, Lleida 25003, Spain; Department of Plant and Environmental Sciences, Clemson University, 105 Collins St., Clemson SC 29634, USA; Department of Plant and Environmental Sciences, Clemson University, 105 Collins St., Clemson SC 29634, USA; Departamento de Producción Animal y Ciencia de los Alimentos, Instituto Agroalimentario de Aragón-IA2 (CITA-Universidad de Zaragoza), C. Miguel Servet 177, Zaragoza 50013, Spain; Departamento de Ciencia Vegetal, Centro de Investigación y Tecnología Agroalimentaria de Aragón (CITA), Avda. Montañana 930, Zaragoza 50059, Spain; Departamento de Producción Animal y Ciencia de los Alimentos, Instituto Agroalimentario de Aragón-IA2 (CITA-Universidad de Zaragoza), C. Miguel Servet 177, Zaragoza 50013, Spain

## Abstract

Sweet cherry is very appreciated by consumers because of its attractive appearance and taste, which is determined by the balanced sweet–sour flavor. In this work, the genetics of soluble solid content (SSC), titratable acidity (TA), sugars, and organic acids was investigated in sweet cherry to facilitate breeding improvement for fruit quality. The fruits of five sweet cherry populations (*N* = 372), three F_1_ and two F_2_, were sampled over two years to evaluate SSC, TA, and the content of individual sugars (glucose, fructose, sorbitol, and sucrose) and organic acids (malic, quinic, oxalic, citric, and shikimic) by ultra-performance liquid chromatography. Glucose, followed by fructose, was the most abundant sugar, while malic acid was the predominant acid. Sorbitol and malic acid were the most stable compounds between years, and had the highest heritability, being also the best correlated to SSC and TA, respectively, revealing their relevance for breeding. Significantly positive correlations were observed among sugars and SSC, and acids and TA, but high interannual variability between years was observed for all traits. Quantitative trait loci (QTL) mapping for SSC, sugars, TA, and organic acids was performed using a multi-family approach with FlexQTL™. Twenty QTLs were detected consistently during the two phenotyped years, and several relevant regions with overlapping QTLs for sugars and acids were also identified. The results confirmed major stable SSC and TA QTLs on the linkage groups 4 and 6, respectively. Within the main LG4 SSC QTL region, where maturity and fruit development time QTLs have been previously detected, three stable sugar (glucose, sorbitol, and sucrose) and two acid (quinic, shikimic) QTLs were also identified, suggesting a pleiotropic effect of ripening date on the content of these compounds. The major malic acid QTL overlapped with TA QTL on LG6; thus, TA QTL mapping on LG6 may correspond to malic acid QTLs. Haplotype analyses of major SSC and sugars QTL on LG4, and TA and malic acid on LG6 revealed haplotypes of breeding interest. Several candidate genes previously identified in other *Prunus* fruit species, like peach, were found to collocate with the QTLs detected herein. This work reports QTLs regions and haplotypes of sugar and acid content in a *Prunus* nonclimacteric stone fruit for the first time.

## Introduction

Sweet cherry (*Prunus avium* L.) is a nonclimacteric temperate fruit tree, which is highly appreciated by consumers, due to its attractive appearance (smooth, red, and shiny), pleasant texture (soft and juicy), and unique flavor (sweet and sour balance). Sweet cherry consumer acceptance is, therefore, based on fruit quality (size, color, texture, and flavor), but is also an important source of nutrients and bioactive compounds [1–3]. The flavor is associated with sweetness and sourness [4–7], which are usually estimated by the balance between soluble solid content (SSC) and titratable acidity (TA). A high ratio between SSC and TA has been related to consumer acceptance [4]. SSC:TA ratios of 19 to 29 are found in most sweet cherry cultivars, with SSC ranging from 15 to 25°Brix, and TA values from 0.7% to 1.2% [8].

Like other fruits, water (80%–83% of the total fruit weight) is the main compound in sweet cherries, followed by carbohydrates (12%–17%). Sugars are the highest portion of carbohydrates, ranging from 11% to 15% of total fruit composition and up to 20% in some cultivars [9]). Sugars contribute to about 65% to 85% of SSC [10]. Glucose and fructose are the main sugars in sweet cherry cultivars, with glucose concentration usually being higher than fructose. Glucose content ranges from 6 to 10 g/100 g of fresh weight (FW), and fructose from 4 to 6 g/100 g FW [11]. Sorbitol and sucrose are also detected but in much lower contents ranging from 0.4 to 4.0 g/100 g FW and 0.05 to 1.18 g/100 g FW, respectively [11]. Fructose has the highest sweetness, followed by sucrose, glucose, and sorbitol [12]. Glucose and fructose accumulate during fruit development, while sucrose and sorbitol accumulation does not exhibit significant changes during ripening [13]. Climatic conditions, rootstock, soil, and agricultural management also influence sweet cherry fruit sugar content [8].

Acidity is another important factor implicated in flavor. The main organic acid in *Prunus* fruits is malic acid. In sweet cherries, its content values range between 360 and 1400 mg/100 g FW depending on the cultivar [9, 14]. Other acids with minor content are citric (5–300 mg/100 g FW) [9, 15], succinic, fumaric, shikimic, and oxalic [11]. In sweet cherry fruit, which is nonclimacteric, malic acid and consequently TA content increase during fruit development [13, 16]. However, in climacteric stone fruit species of the same genus (*Prunus*), like peach [*P. persica* (L.) Batch], plum (*P. salicina* Lindl.), or apricot (*P. armeniaca* L.), malic acid and TA decrease during ripening. Other less abundant acids such as citric or succinic do not have significant content variation during fruit development in sweet cherry [13]. The acidity variation of fleshy fruit is mainly due to the metabolism of malate and citrate in the fruit itself [17]. Several processes are involved in sugar and organic acids accumulation, with carbohydrate transport by the phloem of into the fruit, sugar metabolism, organic acid metabolism, and solute accumulation in vacuoles being the most relevant [17]. About 85% of the sugars required for sweet cherry fruit development are imported from other parts of the plant where they are synthesized [18].

Genetic studies of sweet cherry fruit quality traits have mostly focused on physical attributes like size, weight, firmness [19, 21–22] and skin and flesh color [23, 24] (reviewed in [25]). Fewer works have focused on fruit acceptance and flavor-related traits like sweetness and/or sourness in sweet cherry. Sugars and organic acid content were initially investigated by studying SSC and TA in an F_1_ sweet cherry population (*N* = 601) for three years [26]. No quantitative trait loci (QTLs) were consistently detected for the three years. However, major SSC QTLs were detected for two years on linkage group (LG) 2, and on LGs 4 and 7 in a single year. For TA, QTLs were detected on LGs 2, 4, and 6 but they were not consistent across years. Quero-Garcia et al. [5] evaluated another F_1_ population (‘Regina’ × ‘Garnet’; *N* = 117) for three years and identified a major SSC QTL on LG3. For TA, relevant QTLs were reported on LGs 1 and 6, in three different years, with 16% and 25% phenotype variance explained (PVE), respectively. Similarly, Calle and Wünsch [28] analyzed SSC and TA QTLs in six sweet cherry populations, for two years, using a multi-population approach (*N* = 406, four F_1_ and two F_2_ populations). The major QTL for SSC was detected for the two years on LG4 with a 22% to 34% PVE range. This QTL region collocated with QTLs detected for fruit development time, maturity date, and fruit firmness, indicating a possible relation or pleiotropic effects among these traits [27]. In the same work, additional stable SSC QTLs were identified on LG3 (PVE = 7%–10%). For TA, the most relevant stable QTL was detected on LG6 (PVE = 15%–22%; [27]). Genetic studies of SSC and TA have also been carried out in other stone fruit species like Japanese plum [28, 29], apricot [30, 31], and peach [32–36]. Interannual variation was observed in the QTLs detected for these traits in these species; however, a main SSC QTL on LG4 was also identified in apricot, Japanese plum, and peach [29–31, 36]. The co-localization of LG4 SSC QTL, with firmness and/or fruit development QTLs, was also observed in peach [33]. More recently sugar and acid content genetics have also been investigated in a Chinese cherry (*P. pseudocerasus* Lindl.; [37]) F_1_ population for two years. A polygenetic model was proposed for sugars, while the regulation of acids was suggested to be controlled by two major QTLs [37].

QTL studies for individual sugars and organic acids in *Prunus* species have been carried out in peach and apricot fruit, both of which are climacteric [32, 33, 36, 38, 39]. Dirlewanger et al. [26] analyzed the main sugars (sucrose, fructose, glucose, sorbitol) and the main acids (malic acid, citric acid, and quinic acid) in one F_2_ peach population for two years. Population distribution for all the compounds was similar in both years; however, significant differences between years were observed for some compounds (malic acid and glucose). Sugar and acid contents were analyzed for an additional year in the same population, and the year effect was detected for all the compounds [33]. Using the three-year means, QTLs for SSC, glucose, and fructose were detected on LG4 [33]. These QTLs were detected in the same region as ripening and fruit development period QTLs [33]. Another sucrose QTL was detected on LG6 (37% PVE), near SSC QTL, and both collocated with a candidate gene (*PRUpe;Vp2*), encoding a vacuolar H + -pyrophosphatase, involved in the establishment of an electrochemical gradient across the vacuole, putatively involved in sugar transport across the vacuolar membrane [33]. QTLs for TA (44% PVE), malic acid (83% PVE), citric acid (39% PVE), and sucrose (29% PVE) were identified in the same region on LG5 [33]. Quilot et al. [39] also investigated sugars in a peach × *Prunus* hybrid (*P. davidiana* × *P. persica*) population for two years. Co-localization of QTLs for glucose and fructose was also observed in this work on LGs 2, 4, and 7 [39]. More recently, Zeballos et al. [36] measured the main sugars and acids, TA, and SSC for four years, reporting the highest correlation for SSC and sucrose. For some compounds, such as glucose, stable QTLs were not detected [36]. QTLs for firmness, SSC, and sorbitol were mapped in the same region on LG4, and the major QTL for glucose and fructose content was detected also on LG4 [36] as reported previously [32, 33, 39]. Also, a stable QTL for TA was detected across three years on LG5 [36] in the same location as previously reported [32, 33]. Another study in apricot evaluated organic acids in an F_1_ population. A major QTL was identified for malate and citrate on LG8, while three QTLs were detected for quinic acid on LG5, 6, and 7 [38].

In this work, we have investigated the genetics of sugars and acid content in the nonclimacteric sweet cherry fruit, with the objective of identifying genetic loci associated with the regulation of these relevant compounds of fruit quality. This work serves as a foundation for identifying genes and markers related to sugar and acid content, with the ultimate goal of supporting breeding and selection of sweet cherry cultivars with better fruit quality. We have identified and quantified the main sugar and organic acid content, as well as SSC and TA, for two years in five sweet cherry populations (three F_1_ and two F_2_ populations) and studied their distribution and heritability. Furthermore, to our knowledge, this is the first report on QTL analyses investigating genetic regulation of these compounds using a multi-population mapping approach in a nonclimacteric *Prunus* species.

## Results

### Phenotyping, heritability, and correlations

SSC, TA, and the sugar and organic acid content evaluation was conducted for two years from mature fruit of individuals (*N* = 245 in 2019; and *N* = 263 in 2021) from the five described sweet cherry populations, and from the parental and ancestor cultivars available (*N* = 10), the results of these analyses are shown in Supplementary Tables S1–S4.

#### S‌SC and sugars

**Figure 1:**
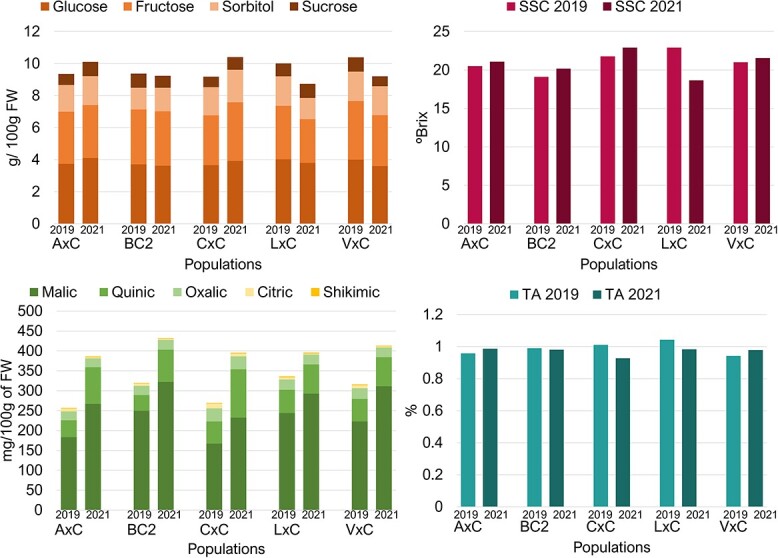
Means of sugars (glucose, fructose, sorbitol, and sucrose) content, soluble solids content (SSC), organic acids (malic, quinic, oxalic, citric, and shikimic) content, and titratable acidity (TA), in populations studied (A × C, BC2, C × C, L × C, V × C), over two years (2019 and 2021).

**Figure 2:**
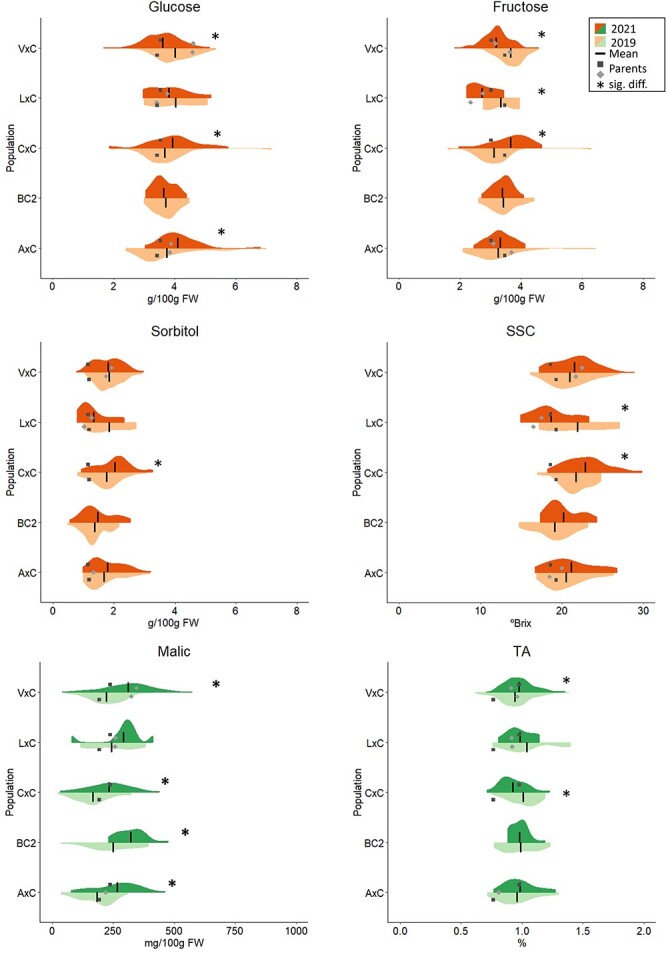
Violin-plot distribution of major sugars (glucose, fructose, and sorbitol) and acids (malic acid), soluble solids content (SSC), and titratable acidity (TA), in each population studied (A × C, BC2, C × C, L × C, V × C) for two years (2019 and 2021). Black vertical lines indicate mean values, squares, and diamonds indicate parental values, asterisks indicate significant differences between year’s means (Student’s test or Mann-Whitney *U*-test; *P*-value < 0.05).

In the ancestor and parental cultivars, SSC values in the two analyzed years varied from 15 (‘Burlat’ in 2021) to 24°Brix (‘Brooks’ in 2021; Table S1). SSC values were consistent for each cultivar between the two years, with interyear variability ranging from 0.5 (‘Van’) to 4.7°Brix (‘Rainier’). ‘Brooks’ had the highest SSC values in both years (around 23 to 24°Brix), while ‘Burlat’, ‘Lambert’, and ‘Rainer’ consistently exhibited the lowest values (15 to 17°Brix) (Table S1). In the population individuals, SSC values showed higher maximum levels than those in the parental cultivars, as individuals with SSC ranging from 15 to 31°Brix were identified (Table S2). The population with the highest SSC mean value was C × C in 2021 (23°Brix), while the lowest mean was observed in L × C in 2019 (19°Brix; Figs 1, 2, and Table S2). SSC means in 2021 were higher than in 2019 (except for L × C), and significant differences between years means were observed in C × C and L × C populations (Student’s test or Mann–Whitney *U*-test, *P*-value < 0.05; Figs 1, 2, and Table S2). Moderate broad-sense heritability (*H^2^* = 0.65) was detected in all the individuals of the five populations. Within populations, SSC heritability ranged from moderate to high, varying from 0.46 (C × C) to 0.65 (V × C) (Table S5). Positive and negative transgressive segregation for SSC was observed in all the populations, except for C × C, which only showed positive transgressive segregation. BC2 parental data (‘BC8’) was not available (Fig. 2).

Four sugars (glucose, fructose, sorbitol, and sucrose) were identified in all the fruit samples of the analyzed cultivars and individuals of the populations. Glucose exhibited the highest content (39%–40% of total sugars), followed by fructose (33%–34%), while sorbitol and sucrose were detected at lower concentrations (15%–18% and 8%–9%, respectively; Fig. 1). For the parental and ancestor cultivars, the levels of glucose and fructose ranged from 2 to 5 g/100 g of FW, while the content of sorbitol and sucrose varied from 0.5 to 2 g/100 g of FW, with the sucrose content being lower than that of sorbitol (Table S1). As for SSC, there was consistent similarity in sugar content between both years within each cultivar, showing interannual variability of less than 0.5 g /100 g of FW for glucose (except for ‘Burlat’), less than 0.8 g/100 g of FW for fructose (except for ‘Burlat’), less than 0.4 g/100 g of FW for sorbitol, and less than 0.2 g/100 g of FW for sucrose. ‘Brooks’, ‘Van’, and ‘Vic’ had the highest content of the four sugars in both years, while ‘Bing’ and ‘Burlat’, in 2021, exhibited the lowest content of glucose and fructose (Table S1).

In the individuals of the populations, glucose and fructose also showed the highest content, with values ranging roughly from 2 to 7 g of glucose, and 2 to 6 g of fructose per 100 g of FW. The content of sorbitol and sucrose ranged from 0.5 to 3.5 and 0.3 to 1.3 g/100 g of FW, respectively (Table S2). The sugar content variation was therefore larger in the population seedlings than in the parental cultivars, as observed for SSC, with C × C and A × C populations showing the largest\variation (Tables S1 and S2). Similarly, significant differences in the mean values of some sugars were observed between years for certain populations. A × C, C × C, and V × C had significant differences between years for glucose, while C × C, L × C, and V × C showed interannual significant differences for fructose (Mann–Whitney *U*-test; *P*-value < 0.05) (Fig. 2). In contrast, sorbitol showed the lowest variation between years, with C × C exhibiting significant differences (Fig. 2 and Table S2). Regarding heritability, glucose and fructose showed the lowest values (*H^2^* = 0.23 and 0.08, respectively), while it was moderately high for sorbitol (*H^2^ =* 0.73). Sugars heritabilities were generally higher when analyzed in each population, except in the C × C (Table S5).

The results show a high interyear variability of the saccharides glucose, fructose, and sucrose, and a higher interyear stability for the sugar alcohol sorbitol and SSC. Only significant positive correlations between years were observed for SSC (*ρ* = 0.41), sorbitol (*ρ* = 0.53), and glucose to a lesser extent (*ρ* = 0.16; Fig. S1), while a significant negative correlation was observed for sucrose. The correlation between sugars and SSC was significantly positive in both years for all sugars (Fig. S1), being moderately high for sorbitol (*ρ* = 0.77/0.84) and glucose (*ρ* = 0.67/0.70), moderate for fructose (*ρ* = 0.47/0.63), and low for sucrose (*ρ* = 0.37/0.14; Fig. S1). Among the sugars, all correlations were also significantly positive in both years. The highest correlations were found between glucose and fructose (*ρ* = 0.86/0.74), as well as between glucose and sorbitol (0.85/0.73) in both years (Fig. S1). This observation, as expected, indicated that glucose, fructose, sorbitol, and SSC increase simultaneously, with sucrose being the sugar that was less correlated with the rest, but still positively correlated.

#### TA and organic acids

In the parental and ancestor cultivars, TA ranged from 0.7% to 1.2% (Table S3). The lowest TA (0.7%) was found in ‘Bing’ (2019) and ‘Burlat’ (2021), and the highest in ‘Van’ (1.2%) in both years (Table S3). Year-to-year variability was generally low (ranging from .0% to 0.2%) except in ‘Bing’, in which variation was 0.7% between years (Table S4). In the individuals of the populations, the TA variability range was like that of parental and ancestor cultivars with values ranging between 0.6% and 1.4% (Fig. 2 and Table S4). TA means were very similar among populations and years, with L × C having the highest mean value (1.0% in 2019) and C × C exhibiting the lowest (0.9% in 2021; Table S4). TA means were higher in 2021 for A × C and V × C; however, C × C and L × C had lower values in 2021 compared to 2019. BC2 presented similar data in both years (Table S4). Significant differences between years were found for TA means in C × C and V × C populations (Student’s test or Mann–Whitney *U*-test, *P*-value < 0.05; Fig. 2). TA showed positive and negative transgressive segregation in all populations, except A × C and C × C in 2019 (Fig. 2). Overall, moderate *H^2^* for TA was observed (*H^2^* = 0.43) for all plant material in the study, whereas within populations heritability values ranged from 0.21 (BC2) to 0.70 (V × C; Table S5).

Five organic acids (malic, quinic, oxalic, citric, and shikimic) were identified in all samples, including parental cultivars and individuals of the populations. Malic was the predominant acid, accounting for an average of 71% of the total acid content in the parental and ancestor cultivars, while the other organic acids were detected at lower concentrations, with quinic accounting for 12%–24%, oxalic 6%–12%, citric 1%–4%, and shikimic acid ≤ 1% (Table S3). In these cultivars, malic content ranged from 89 to 433 mg/100 g of FW, followed by quinic (29 to 109 mg/100 g of FW), oxalic (10 to 28 mg/100 g of FW), citric (0.6 to 9 mg/100 g of FW) and shikimic (1 to 4 mg/100 g of FW) (Table S3).

Malic acid was also the most abundant acid in the individuals of the populations, with values ranging from 24.7 to 575.4 mg/100 g of FW and accounted for 59% to 78% of total acid contents (Fig. 1 and Table S4). C × C population had the lowest malic content mean (167 mg/100 g of FW in 2019), while BC2 had the highest (313 mg/100 g of FW in 2021; Table S3). The range of quinic and oxalic content in the populations was broader than those of the parental and ancestor cultivars (Tables S3 and S4). Quinic content ranged from 19 to 165 mg/100 g of FW, and oxalic content varied from 7 to 53 mg/100 g of FW. Citric acid showed a wide range (0.8–24 mg/100 g FW), as observed in the parental and ancestor cultivars (Tables S3 and S4). Positive and negative transgressive segregation was shown for malic acid in all populations (Fig. 2). Interannual variability was observed for this acid with significantly different means detected in all populations except in L × C (Student’s test or Mann Whitney *U*-test, *P*-value < 0.05; Fig. 2). Overall, malic acid had the highest heritability (*H^2^* = 0.58), followed by oxalic and shikimic acid (*H^2^* = 0.49 and 0.41, respectively). Quinic acid showed a moderate-low *H^2^* (0.32) and citric acid had the lowest (*H^2^* = 0.15). A wide range of heritability was observed for the different organic acids in each population, with specific acids exhibiting higher heritabilities in each population (Table S5).

TA and organic acids showed even higher interyear variability than sugars, with a significantly low-moderate positive correlation for TA (*ρ* = 0.29), malic (*ρ* = 0.33), oxalic (*ρ* = 0.32), and shikimic (*ρ* = 0.27) acids, and low for quinic (*ρ* = 0.19), and no significant correlation between years for citric acid (Fig. S1). A moderately significant positive correlation was found between malic acid and TA in both years (*ρ* = 0.45/0.47; Fig. S1), being the highest correlation of all the organic acids with TA. Among acids, the highest correlations were shown between quinic and shikimic (*ρ* = 0.52/0.58) and quinic and oxalic (*ρ* = 0.46/0.60; Fig. S1).

### QTLs analyses

#### S‌SC and sugars

**Figure 3:**
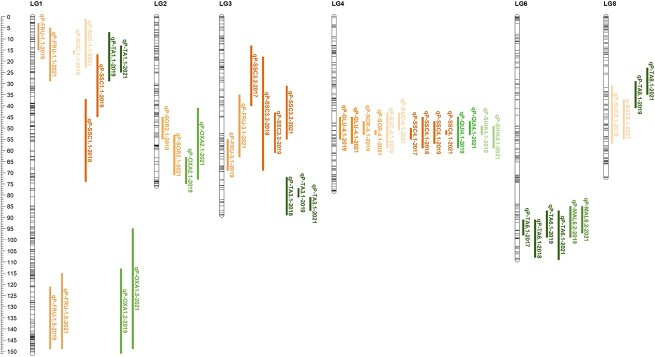
Genetic position (cM) on the consensus linkage map [27] of stable and significant QTLs of SSC and TA (Table 1), sugars (glucose, fructose, sucrose, and sorbitol; Table 2), and acids (malic, quinic, oxalic, citric, shikimic; Table 2) QTLs.

A total of 57 QTLs were detected for the analyzed sugars and SSC (Table S6). From these, 24 were detected in 2019 and 33 in 2021. Among these, sixteen had strong evidence (average *2lnBF* > 5) and one decisive evidence (average *2lnBF* > 10; Table S6). Stable QTL regions, with overlapping QTLs detected in each year, were identified for SSC and the four sugars, glucose, fructose, sorbitol, and sucrose, on LGs 1, 3, 4, and 8 (Tables 1, 2; Table S6; and Fig. 3). The most significant SSC QTL, *qP-SSC4.1^m^,* was detected in a narrow region on LG4 (51 to 53 cM; Table 1). This QTL had strong evidence and explained a significant amount of the phenotypic variance (PVE), 33% and 38% in 2019 and 2021, respectively (Table 1). Another SSC QTL, *qP-SSC3.1^m^*, was detected on LG3 (31–64 cM) (Table 1) with decisive evidence in 2021. The *qP-SSC3.1^m^* exhibited a smaller effect in 2019, indicating reduced stability in controlling SSC (Table 1).

**Table 1:** SSC and TA stable QTLs (detected at least two years), and significant (Average 2lnBF > 2), from four-year data (2017 and 2018 data from [27]; 2019 and 2021 data from this work).

	**LG**	**Year**	**QTL name**	**Interval (cM)**	**Peak (cM)**	**Max2lnBF**	**Average 2lnBF**	**Additive effect**	**PVE (%)**	**Physical position in sweet cherry genome (Mbp)**
	1	2018	*qP-SSC1.1^m^*	37–74	63	9.01	4.82	1.26	15.26	10.4–17.2
	1	2019	*qP-SSC1.1^m^*	17–45	33	3.66	2.73	0.61	3.58	14.0–35.8
	3	2017	*qP-SSC3.2^m^*	13–40	27	9.32	4.76	1.5	10.42	8.1–17.5
	3	2018	*qP-SSC3.2^m^*	18–69	59	8.29	4.19	0.89	7.41	9.1–29.3
	3	2019	*qP-SSC3.2^m^*	43–61	45	4.6	2.92	0.37	0.61	17.6–25.3
	3	2021	*qP-SSC3.2^m^*	31–55	35	6.58	4.15	0.87	11.9	12.5–23.2
	4	2017	*qP-SSC4.1^m^*	50–55	53	14.09	11.7	3.04	34.16	14.8–17.4
	4	2018	*qP-SSC4.1^m^*	45–59	53	9.6	6.82	1.69	22.11	13.1–17.9
	4	2019	*qP-SSC4.1^m^*	47–57	51	10.94	7.05	1.52	32.8	14.0–17.6
**SSC**	4	2021	*qP-SSC4.1^m^*	51–53	51	11.25	9.55	2.08	38.07	15.1–16.3
	1	2019	*qP-TA1.1^m^*	7–29	17	6.23	3.82	0.07	5.26	6.9–10.4
	1	2021	*qP-TA1.1^m^*	13–25	25	3.26	2.56	0.05	<1	8.4–12.1
	3	2018	*qP-TA3.1^m^*	72–89	87	9.94	6.17	0.06	5.01	30.5–36.1
	3	2019	*qP-TA3.1^m^*	77–81	81	2.78	2.03	0.03	<1	31.5–32.8
	3	2021	*qP-TA3.1^m^*	81–87	85	3.77	3.46	0.07	<1	32.8–34.9
	6	2017	*qP-TA6.1^m^*	91–98	95	11.83	9.65	0.09	21.57	33.1–33.8
	6	2018	*qP-TA6.1^m^*	91–108	97	10.29	6.33	0.07	15.02	33.1–36.9
	6	2019	*qP-TA6.1^m^*	87–99	95	6.01	4.33	0.05	<1	31.5–34.0
	6	2021	*qP-TA6.1^m^*	87–109	95	8.99	5.18	0.05	7.14	31.5–37.4
	8	2019	*qP-TA8.1^m^*	29–41	31	3.53	2.66	0.06	<1	23.2–27.0
**TA**	8	2021	*qP-TA8.1^m^*	23–35	27	2.78	2.18	0.04	<1	21.8–24.5

QTLs interval in cM, maximum 2ln Bayes Factor (2lnBF), average 2lnBF, mean additive effect, percentage of variance explained (PVE), and physical position in the Tieton cv. Genome v2.0 [40] are shown.

**Table 2:** Sugars and organic acids stable QTLs (detected at least two years), and significant (Average 2lnBF > 2) from two-year data (2019, 2021).

	**LG**	**Year**	**QTL name**	**Interval (cM)**	**Peak (cM)**	**Max 2lnBF**	**Average 2lnBF**	**Additive effect**	**PVE (%)**	**Physical position in sweet cherry genome (Mbp)**
Glucose	4	2019	*qP-GLU4.1^m^*	45–55	51	9.71	7.18	4.08	25.22	14.0–17.4
(GLU)	4	2021	*qP-GLU4.1^m^*	45–57	53	11.38	6.49	3.38	24.07	14.0–17.7
Fructose	1	2019	*qP-FRU1.1^m^*	3–15	11	4.27	3.91	1.95	4.06	5.8–9.0
(FRU)	1	2021	*qP-FRU1.1^m^*	5–29	21	6.8	3.78	0.96	3.4	6.5–12.9
	1	2019	*qP-FRU1.5^m^*	121–149	141	4.09	2.49	2.14	2.75	49.9–57.2
	1	2021	*qP-FRU1.5^m^*	115–149	129	4.465	1.86	3.262	4.73	47.7–57.2
	3	2019	*qP-FRU3.1^m^*	55–69	59	5.97	4.57	2.77	8.14	21.5–29.3
	3	2021	*qP-FRU3.1^m^*	35–63	45	7.92	5.31	1.15	6.08	14.3–26.6
Sorbitol (SOR)	2	2019	*qP-SOR2.1^m^*	41–55	47	9.28	6.17	2.68	28.65	32.6–36.8
	2	2021	*qP-SOR2.1^m^*	53–71	63	6.05	4.67	2.73	12	36.5–39.7
	4	2019	*qP-SOR4.1^m^*	47–53	51	12.21	8.53	3.42	27.48	14.0–16.3
	4	2021	*qP-SOR4.1^m^*	51–53	53	12.17	11.77	5.32	51.45	14.0–16.3
Sucrose	1	2019	*qP-SUC1.1^m^*	15–17	16	2.95	2.95	0.36	<1	9.0–10.4
(SUC)	1	2021	*qP-SUC1.1^m^*	1–23	1	5.14	2.72	0.6	1.96	4.9–11.4
	4	2019	*qP-SUC4.2^m^*	43–59	49	6.16	4.44	0.38	4.41	14.0–18.6
	4	2021	*qP-SUC4.2^m^*	43–51	45	3.11	2.28	0.19	<1	14.0–15.1
	8	2019	*qP-SUC8.1^m^*	31–57	33	4.75	2.953	1.7	7.12	23.2–31.34
	8	2021	*qP-SUC8.1^m^*	37–55	55	6.63	3.89	0.94	7.3	26.1–31.14
Malic	6	2019	*qP-MAL6.2^m^*	85–99	95	10.04	6.68	37.57	11.11	31.0–33.6
(MAL)	6	2021	*qP-MAL6.2^m^*	85–97	95	12.36	6.001	49.837	13.7	31.0–34.0
Quinic	4	2019	*qP-QUI4.1^m^*	45–59	53	9.26	7.40	10.22	16.67	14.0–17.9
(QUI)	4	2021	*qP-QUI4.1^m^*	47–53	51	12.43	5.76	17.38	19.34	14.0–16.2
Oxalic	1	2019	*qP-OXA1.3^m^*	113–151	147	5.61	4.07	5.89	8.26	47.3–55.8
(OXA)	1	2021	*qP-OXA1.3^m^*	95–149	137	5.80	4.21	5.30	21.13	42.3–57.3
	2	2019	*qP-OXA2.1^m^*	63–75	69	3.26	2.94	4.29	1.81	38.7–44.1
	2	2021	*qP-OXA2.1^m^*	41–73	69–71	4.27	2.81	4.02	3.64	32.7–40.5
Shikimic	4	2019	*qP-SHIK4.1^m^*	49–53	51	12.58	8.56	0.69	<1	14.8–16.2
(SHIK)	4	2021	*qP-SHIK4.1^m^*	49–53	51	11.27	7.20	0.36	23.07	14.8–16.2

QTLs interval in cM, maximum 2lnBF, average 2lnBF, mean additive effect, percentage of variance explained (PVE), and physical position in the Tieton Genome v2.0 [40] are shown.

For the individual sugars analyzed, the most relevant QTLs were detected for glucose (*qP-GLU4.1^m^*) and sorbitol (*qP-SOR4.1^m^*) in the same narrow LG4 region as for SSC (45–57 cM). These QTLs showed strong to decisive evidence and PVE ranging from 24% to 51% (Table 2 and Fig. 3). These LG4 QTLs were the only stable QTLs detected across both years for glucose and sorbitol. Another QTL was detected for sucrose (*qP-SUC4.2^m^*) in the same region with lower significance, but not for fructose. QTLs for sucrose were detected on LGs 1 (*qP-SUC1.1^m^*; 1–23 cM) and 8 (*qP-SUC8.1^m^*; 31–57 cM). For fructose, two QTLs were identified on LG1: *qP-FRU1.1^m^* (3–29 cM), which overlaps with the sucrose QTL (*qP-SUC1.1^m^*), and *qP-FRU1.5^m^* (115–149 cM), which overlaps with the oxalic QTL (*qP-OXA1.3^m^*). Another QTL for fructose was detected on LG3 (*qP-FRU3.1^m^*; 35–69 cM; Fig. 3, Tables 1 and 2) overlapping with SSC QTLs. Several relevant genomic regions were observed where various overlapping QTLs for SSC and/or sugars were mapped (both or either year), with variable degrees of significance (Fig. S2). These regions are located on upper and lower LG1 (1–25 and 115–149 cM), and lower LG2 (41–73 cM), LG3 (35–36 cM), and LG4 (43–59 cM) (Fig. S2).

#### TA and organic acids

A total of 55 QTLs were detected for the analyzed organic acids and TA (Table S7). Of these QTLs, 31 and 24 were detected in 2019 and 2021 respectively; with 16 of them reported with strong evidence (average *2lnBF* > 5; Table S7). Overlapping QTLs, detected in both years, were mapped for different organic acids (malic, quinic, oxalic, and shikimic) and TA on LGs 1, 2, 3, 4, 6, and 8 (Tables 1 and 2, and Fig. 3).

For TA, the most significant QTL, *qP-TA6.1^m^*, was detected in a narrow region (87–109 cM) on LG6 with strong and decisive evidence in 2019 and 2021, respectively (Table 1 and Fig. 3). Other TA QTLs were detected on LGs 1, 3, and 8 (Table 1 and Fig. 3). None of these TA QTLs explained a large proportion of the phenotypic variance (PVE < 7.1). For malic acid, the major organic acid, a stable QTL, *qP-MAL6.2^m^* (85–99 cM) was detected overlapping with the major TA QTL on LG6. This QTL explained 11% to 13% of PVE (Table 2 and Fig. 3). However, none of the other detected QTLs for malic acid in both years overlapped with TA QTLs. For quinic acid, one QTL, *qP-QUI4.1^m^* was detected on LG4 (45–59 cM; PVE 16–19), in the same region where a QTL for shikimic acid (*qP-SHIK4.1^m^*) was found. This region overlapped with the genomic area where several QTLs for sugar were also found. Another two QTLs were detected for oxalic acid on LGs 1 and 2 (*qP-OXA1.3^m^*, *qP-OXA2.1^m^*), with the former showing strong evidence (Table 2 and Fig. 3). For citric acid, no stable QTLs were detected across years. However, two QTLs with strong evidence were observed in 2019, and four in 2021, on LGs 1, 2, 3, and 4 (Table S7).

### Haplotype analyses of breeding interest

The haplotypes for the main SSC, glucose, sorbitol, quinic, and shikimic acid QTLs on LG4, and for TA and malic acid QTLs on LG6 were constructed for the parental and ancestor genotypes (Tables S8 and S9). On the LG4 QTLs, within 56–59 cM, the four haplotypes (*H4-a, −b, −c,* and *-d*) were identified with six SNPs (Table S8). *H4-c* haplotype, which was exclusively identified in ‘Cristobalina’, ‘Burlat’, and ‘BC8’, exhibited the lowest values of SSC sugars and acid content (Tables S8 and S10). Conversely, the *H4-a* haplotype was associated with a higher SSC and sugars (glucose and sorbitol), and acids (quinic and shikimic) content (Table S10). The comparison of means within the *H4* haplotypes revealed that *H4-b* exhibited higher values than *H4-c* and *-d*.

On the LG6, within the 95–96 cM interval, where TA (*qP-TA6.1^m^)* and malic (*qP-MAL6.2^m^)* acid QTLs were identified, four SNPs were used for haplotype construction. Three haplotypes were identified (*H6-a, −b,* and *-c*) in parental and ancestor genotypes (Table S9). Significant differences among the mean TA values were observed in both years, with the *H6-c* showing the highest values (Table S11). For malic acid, significant differences were detected only in one of the studied years; however, the *H6-c* haplotype also exhibited the highest mean values in both years (Table S11).

## Discussion

Genetics and interannual variability of individual sugars and organic acids in sweet cherry were analyzed for two years, in five populations (including F_1_ and F_2_), with the goal of carrying out QTL analyses that could be useful for sweet cherry fruit quality breeding. In parallel, SSC and TA were also analyzed in the same samples, adding two-year data to previous study ([27]; Table 1), to help understand their genetics, and correlation between sugars and acid content. Similar works, including sugar and/or acid phenotyping followed by genetic and QTL analyses, have only been carried out previously in *Prunus* species with climacteric fruits such as peach and apricot [32, 33, 38, 39].

### S‌SC and sugars

#### Phenotyping heritability and correlations

In this work, the range of SSC observed in the populations (15–31°Brix) was broader than previously observed in sweet cherry cultivars (12–24°Brix) [1, 11]. However, this variability was similar to that previously observed (13 to 30°Brix) in the same plant material for two additional years (2017 and 2018; [27]). In addition, SSC heritability (0.65) was very similar to that previously estimated for this plant material (0.62; [27]), and higher than that observed in other sweet cherry populations (*H*^2^ = 0.48; [41]). In Chinese cherry, larger variability of SSC heritability in different years (0.14–0.94; [37]) was reported. However, heritability estimation was done differently from this study so results could not be compared. Studies in other *Prunus* species like peach, show similar SSC heritability than reported here (*H*^2^ = 0.72–0.76) [35, 42]. These results indicate significant variability in SSC within the analyzed plant material, as well as evidence of genetic inheritance, highlighting the potential for improvements in sweet cherry via breeding.

For the analyzed sugars, glucose and fructose content (5–7 g /100 g FW) accounted for about 70% of the total sugar content, which is in agreement with previous studies, although slightly higher and lower values have been observed in other sweet cherry cultivars [2, 14, 43, 8, 9, 11]. The sorbitol and sucrose were found at lower contents (0.1–4 /100 g FW) than glucose and fructose but within the ranges previously described [8]. In the related Chinese cherry, a broad range of variation in major sugars was also observed [37], but with fructose as the major sugar, while glucose had a higher contribution to total sugar content in this work. In peach, the major reported sugar is sucrose [32]. The differences in these species may be revealing intrinsic differences of the fruit quality of each species and/or a different genetic variability for these traits. The heritability for individual sugars has been estimated in peach with similar results as reported in this work: high heritability for sorbitol and SSC, and moderate *H^2^* for glucose and fructose [42]. Nevertheless, when the heritability was analyzed across populations, variations were noted among different sugars in each population. Some populations exhibited higher heritability than others for specific sugars, suggesting that genetic background influences sugar heritability.

The results showed a low interyear correlation and hence a high annual variability for all sugars except for sugar alcohol, sorbitol which was the most stable sugar between years. Similar results have also been shown previously in peach with sorbitol being more stable than other major sugars [36], and in Chinese cherry [37] in which major sugars were also highly variable between years. This year-to-year variability is linked to environmental factors such as temperature, radiation, and water supply, which influence the metabolic regulation of these compounds [44]. Significant differences were observed in temperature and precipitation in both sampling seasons (Fig. S3). In 2019, precipitation was concentrated earlier whereas in 2021, it was distributed throughout the ripening and harvest season. Temperatures were quite similar during the ripening time both years, except at the end of the harvest season with higher temperatures recorded in 2021. These differences may account for variability in compound concentration the different years and identification of minor QTLs only one year.

Also, in this work, the highest correlation was observed between SSC and sorbitol, followed by glucose and fructose. However, in Chinese cherry, the highest correlation was observed between SSC and the main sugars glucose and fructose [45]. The results reported here reveal that sugar alcohol sorbitol, despite not being the major sugar, is the most stable component of SSC in sweet cherry. Sorbitol was also better correlated with SSC, and with the highest heritability, being therefore a relevant candidate trait for sweet cherry breeding fruit quality improvement in the species.

#### A major QTL on LG4 for SSC and sugar content

A major stable SSC QTL was identified on LG4, *qP-SSC4.1^m^* in both phenotyped years. This same SSC QTL was detected previously in the same plant material with similar PVE (22–34; [27]). This LG4 region overlaps with SSC QTLs identified in other *Prunus* species [5, 29, 36]. Additionally, the major stable QTLs for glucose and sorbitol content (*qP-GLU4.1^m^, qP-SOR4.1^m^*) detected in this work also overlap with this major SSC QTL on LG4 and explained more than 24% of the phenotypic variance (up to 50% for sorbitol). These results confirm the importance of LG4 region in SSC regulation and reveal its crucial role in controlling glucose and sorbitol content in sweet cherry. These two sugars are highly correlated with SSC and play a major role in sweet cherry quality being the major and most stable sugars, respectively, as discussed above. In this LG4 genomic region, García-Gómez et al. [30] identified three candidate genes *ppa001122m, ppa000854m*, and *ppb001660m* associated with SSC regulation. By BLAST analysis ([46]; www.rosaceae.org) using the ‘Tieton’ sweet cherry genome as reference (v2.0; [40]), we confirmed the presence of orthologous genes (*FUN_033560*, *FUN_033567*) for two of these candidate genes (*ppa001122m, ppa000854m*) in the LG4 QTL region associated to SSC. Additionally, in the same region, we identified an orthologous gene in sweet cherry (*FUN_034033*) for another candidate gene associated with sorbitol regulation (*Prupe.4G191900*; [47]) that was previously identified in peach using the same analyses. This gene encodes a diacylglycerol kinase 5 (DGK5), which was expressed during fruit development in peach [47]. These candidate genes for sugar regulation may also be playing a relevant role in sugar content regulation in sweet cherry.

The same LG4 region also overlaps with major QTLs for maturity date, fruit development time, and firmness in sweet cherry, being, therefore, a highly relevant hotspot for fruit quality breeding in sweet cherry [27]. In peach, QTLs for individual sugars have also been detected within the same LG4 region previously associated with maturity date [33, 36, 39]. Moreover, this QTL region on LG4 has been compared between sweet cherry and peach, where homologous genes controlling key processes related to ripening time and fruit firmness were identified [48]. This conserved region has also been found in other *Prunus* species, including apricot [28] and Japanese plum [29], confirming the genetic regulation of maturity and sugar accumulation within the same genomic region on chromosome 4. The conservation of this region extends beyond the *Prunus* genus to other Rosaceae species. A syntenic region corresponding to peach LG4 was identified on LG10 in apple, where QTLs for SSC, maturity, and sugars were also detected [49, 50]. Other authors have also highlighted the importance of this region in terms of fruit maturity and sugar content, suggesting the possible pleiotropic effect of maturity on other fruit quality traits like sugars, in both peach and sweet cherry [27, 51, 52]. In sweet cherry, Calle and Wunsch [27] demonstrated a strong correlation between SSC, maturity date, and fruit development period using the same plant material and haplotype analyses in this region. They observed that haplotype *H4-c*, which had a shorter fruit development period and earlier maturity date, also showed lower SSC content. In this work, the analysis of the same haplotypes in this LG4 region revealed lower values for SSC and sugar content (glucose and sorbitol) in the same haplotype (*H4-c*), but also lower values of quinic and shikimic acids. These findings further highlight the relationship between the ripening process and the accumulation of sugars and acids suggesting the possibility that genes associated with ripening could have a pleiotropic effect on the regulation of sugars concentration in the species.

From the breeding perspective, selection of haplotypes *H4-a* and *H4-b* can contribute to higher SSC and sugar content than *-c* or *-d*. Therefore, selection of the earlier maturity haplotype *H4-c* will also involve decreasing sugar content as shorter development period is correlated with lower SSC and lower sugar content as shown herein. Minor QTLs may play a relevant role in quality fruit breeding with the potential to serve as a resource for increasing sugar content without prolonging the fruit development period and delaying the ripening date.

##### Other relevant genomic regions involved in SSC and sugar content.

Another stable SSC QTL was identified on LG3 (*qP*-*SSC3.1^m^*), which explains a lower portion of the phenotypic variation than *qP-SSC4.1^m^*, and was also detected previously in the same material [27]. Additionally, a QTL for fructose (*qP-FRU3.1^m^*) detected in two years, along with two QTLs for glucose (*qP-GLU3.1)* and sorbitol (*qP-SOR3.1*) reported uniquely in 2021, were identified in this same region. In this LG, an SSC QTL was also previously found in sweet cherry, although it is unclear if in the same genomic region as in this work [5]. However, it is evident that region on LG3, is relevant for and playing a stable role in SSC and sugar content regulation.

For fructose, two stable and significant QTLs, *qP-FRU1.1^m^* and *qP-FRU1.5^m^* were detected on LG1. The first one was colocalized with QTLs for sucrose and sorbitol (*qP-SUC1.1^m^, qP-SOR1.1*) in this work. The second, which is located at the bottom of LG1, encompassed the region where two genes (*LOC110761288*, *LOC110744941*) related to sugar metabolism in sweet cherry were previously reported [53]. We identified two orthologous genes (*FUN_006074* and *FUN_007451*), in the ‘Tieton’ genome (v2.0; [40]). The *FUN_006074* was annotated as ATP-dependent 6-phosphofructokinase 3 and the *FUN_007451* as probable receptor-like protein kinase.

For sucrose, the stable QTL with the highest PVE was found on LG8. However, the main sucrose QTL in peach is found on LG5, overlapping with other main SSC QTLs [23, 36]. QTLs in this LG5 region were not detected in sweet cherry for SSC or sucrose. As sucrose is a minor sugar in sweet cherry, opposite to peach in which it is the most abundant, it may be more difficult to identify SSC QTLs associated with sucrose regulation in sweet cherry, or this region may not be segregating in our population due to a lower variability in sweet cherry.

The distribution of QTLs involved in regulating sugar content across all LGs indicates that the genetic control of these traits is dispersed throughout the genome. This pattern is consistent with previous observations in peach [33, 39] and recent findings by Ma et al. [37] in Chinese cherry, that suggest that sugar content is regulated polygenically with additive effects. Furthermore, the influence of environmental conditions in different years during the fruit ripening process could be associated with the varying percentages of variance explained by certain minor QTLs, as well as the identification of these QTLs in one year but not in another. These genotype-by-environment and QTL-by-environment interactions have been observed in the species during QTL analyses of fruit-related and agronomical traits [19, 54]. The clustering of many of these sugar QTLs in different regions of the genome has also been reported previously in peach [33, 36] and [55]. The overlapping of QTLs for different sugars in the same genomic regions would be consistent with the common sugar metabolic pathways in *Prunus* [10].

### TA and organic acids

#### Phenotyping, heritability, and segregation

The TA values were similar to those previously reported for sweet cherry cultivars (0.6%–1.4%; [14]). However, the TA means were higher than those found by [27] in the same plant material. The observed variation in the mean values may be due to maturation degree or environmental effect. The contrasting environmental conditions between the two seasons were clearly discernible, as illustrated in the graph (Fig. S3) and discussed above. Heritability for TA (0.43) was lower than previously reported in the same materials during two different years (*H^2^* = 0.54; [27]). However, when heritability was calculated for each population, it ranged from 0.21 to 0.70, depending on the population. Thus, the results highlight the high impact of annual environmental and population genetics on TA variability, with the ripening stage at sampling potentially also playing a role [16]. The TA heritability was similar to that reported in other sweet cherry populations (0.60; [34, 41]), and generally lower than in peach (0.90–0.93; [35]) confirming that sweet cherry fruit TA heritability may be lower than that of other *Prunus* species like peach. In peach, TA is controlled by a major locus on LG5 (*D* locus; [56]), which contrasts with sweet cherry where TA is controlled by multiple minor genes, potentially resulting in reduced heritability.

The values for malic acid (predominant acid) were lower than previously described for this acid in other plant materials (300–1100 mg/100 g for FW; reviewed in [1]). The *H^2^* for this acid was 0.58, slightly lower than that reported for the same compound in apricot (*H^2^* = 0.79; [29]). However, when considering the *H^2^* for malic acid in each population individually, significant variation was observed, highlighting the influence of genetic background on the segregation of this acid. The second most abundant acid, quinic acid, has not been quantified in most of the previous studies [2, 9, 16], although it has been detected at low concentrations [57]. However, the oxalic acid was found at higher concentrations than previously reported (< 5 mg/100 g of FW; [8]), and the shikimic acid content was comparable to levels observed in certain cultivars [14], but lower than in others [2]. The citric acid showed a wide range as described by the bibliography [34].

Low and moderate interannual correlations of organic acids and TA confirmed the high interannual variability of these parameters in this plant material and may be attributed to environmental effects or the ripening stage in which were harvested, as discussed above. As mentioned above, there were notable differences in temperature and precipitation between the two years analyzed (Fig. S3). The correlations among acids were significantly positive in this work as observed in peach [32, 39], but not in Chinese cherry [45]. Amongst them, the highest correlation was observed between malic acid and TA as previously shown in Chinese cherries and peach [32, 39, 45], and confirming the large contribution of malic acid, the most abundant acid in cherries to TA.

#### A major stable QTL for TA and malic acid on LG6

The main QTL for TA, *qP-TA6.1^m^*, was detected on LG6 in the same position previously described for the same plant material in two different years (2017 and 2018; [27]). This confirms the detection of this QTL over four years, making it the only QTL consistently identified across all four years. This QTL explains a relevant portion of the TA variation (15%–21%), and it is located in a syntenic region where QTLs for TA have also been found in peach [34] (Chr06: 12.07–37.69 Mbp in the ‘Tieton’ genome v2.0 and Chr06: 8.88–30.72 Mbp in the peach genome v2.0.a1; https://www.rosaceae.org/synview/block/ppptB235). However, the main QTL for TA in peach was detected on LG5 [35, 36], a region where no QTLs were detected in this work in sweet cherry. Furthermore, the primary QTL for malic acid (*qP-MAL6.2^m^*), the predominant acid detected in sweet cherry, was identified in the same region as *qP-TA6.1^m^*. This QTL has a large additive effect although it only explains 11%–13% of the variation. However, the strong correlation between malic acid and TA may explain the colocation of their QTLs, suggesting that the regulation of TA in this region is caused by the malic acid QTL. The haplotype analysis confirmed this hypothesis, since haplotype *H6-c* that increases TA level was observed to increase malic acid content as well. Consequently, cultivars such as ‘Burlat’ or ‘BC8’ homozygous for this haplotype can be used to increase fruit acidity and should be avoided if the breeder’s purpose is to reduce acidity, in accordance with market demands.

In this LG6 region, we identified a candidate gene, described as a vacuolar-type inorganic pyrophosphatases (V-PPase) (*FUN_022609*), using the ‘Tieton’ genome v2.0 [40]. Several studies indicated that V-PPase is involved in the accumulation of sugar and organic acids in the vacuole during the fruit development in pear (*Pyrus communis* L.; [58]), Japanese pear (*P. serotina*; [59]), grape (*Vitis vinifera* L.; [60]), peach [33], and tomato [61], making it a good candidate gene for the phenotypic variation explained by this QTL.

#### Other relevant genomic regions involved in TA and organic acids regulation

Three other relevant and stable regions (detected in 2 or 3 years) for TA regulation were identified on LGs 1, 3, and 8. Of these, TA QTLs on LG1 overlapped with stable QTLs for oxalic acid on LG1, but not with other stable (at least in two years) QTLs for other acids. As in malic acid on LG6, this TA QTL on LG1 may be caused by the oxalic acid regulation in that region. In contrast, the main QTLs for quinic and shikimic acids (*qP-QUI4.1^m^, qP-SHIK4.1^m^*) were found to overlap with the main region described for SSC and sugars regulation on LG4, but not for TA regulation. The colocalization between QTLs for sugars and TA has also been previously described in peach [39]. These findings may suggest the potential co-regulation of these compounds and/or their association with the regulation of ripening, as the ripening date has been previously shown to be regulated in a large portion on the same region of LG4 [27].

The high number of QTLs detected for TA and organic acids in this work contrasts with the results observed in peach [35, 36], in which QTLs on LG5 play a major role, and in Chinese cherry [37] in which two pairs of additive-dominant major genes were proposed to regulate acidity and malic acid content. Similarly, two main QTLs for the regulation of TA and malic acid were observed in apple (*Malus domestica* Borkh; [62]. In this work, QTL analyses revealed a larger number of genomic regions playing smaller roles in organic acids and hence TA regulation in sweet cherry.

In this work, genomic regions associated with sugar and organic acid content were identified, which contribute to sweetness and acidity in cherries and provide valuable insights into breeding programs to improve the fruit organoleptic characteristics. Even though several factors like the environment and the ripening stage highly influence the levels of sugars and organic acids in the fruit, the wide segregation of these compounds within populations and the presence of stable QTLs over the two studied years provide evidence of the possibility of selection for improvement of these traits. The major QTL regions and hotspots identified here may help in developing breeding tools for sweet cherry quality breeding, a fruit that reveals differences in sugar and acid content with other climacteric stone fruits. Additionally, to further explore the genetic control of these compounds and identify and validate candidate genes within the main QTL intervals, transcriptomic analyses of sweet cherry fruit at various ripening stages are being carried out.

## Materials and methods

### Plant material

The plant material used in this work includes five sweet cherry populations (*N* = 372) and available parental cultivars and ancestors (*N* = 10; Table S1). These five populations include three cross-pollinations (F_1_), namely ‘Lambert’ × ‘Cristobalina’ (L × C; *N* = 14), ‘Vic’ × ‘Cristobalina’ (V × C; *N* = 158), and ‘Ambrunés’ × ‘Cristobalina’ (A × C; *N* = 40); and two populations derived from self-pollination (F_2_), one from the cultivar ‘Cristobalina ʼ (C×C; *N* = 97) and the other from the selection ‘BC-8’ (BC2; *N* = 68). These populations are maintained in the experimental orchards of CITA de Aragón (Zaragoza, Spain).

### S‌SC, TA, sugars, and organic acid content quantification

A total of 249 and 263 individuals were sampled in 2019 and 2021, respectively (Table S2). A representative count of fruits (15 fruits per tree) was collected from each individual over two years. The fruit samples were harvested based on commercial maturity, using a combination of colour, firmness, and taste assessment. The fruit samples were pitted and stored at −20°C. For further analyses, fruit samples were defrosted and homogenized using a homogenizer (POLYTRON ® KINEMATICA; Malters, Switzerland). SSC was measured from the fruit homogenate using a digital refractometer (PAL-1, Atago, Tokyo, Japan). TA was determined by dissolving 5 g of the same homogenate in 50 ml of de-ionized water using an automatic titrator (Metrohm, Herisau, Swiss).

Sugars and organic acids were extracted from the same samples and measured by ultra-performance liquid chromatography (UPLC). Sugar and acid extractions were carried out using an adaptation of the method described by Sturm et al. [63]. Five grams of homogenate and 20 ml of ultrapure water (MiliQ) were mixed and shaken for 1 min using a vortex and another minute using a homogenizer. Samples were subsequently treated with ultrasound using a sonicator for 5 minute (BACTOSOMIC 14.2, Bandelin, Berlin, German). The solution was then centrifuged for 20 minute at 9500 rpm. The pellet and supernatant were separated by decantation. The pellet was used in a second extraction by adding 10 ml of MiliQ water and repeating the steps described above from the vortex mixture step. The supernatants of the two extractions were mixed and centrifuged one more time under the same conditions, and the liquid phase obtained was used for the sugar and acid extraction. For the sugars, 2 ml of this extraction (liquid phase) was filtered with a 1.0/0.45-μm polyester (PET) double syringe filter followed by another filtration through a 0.20-μm filter. For the acids, 3 ml of the extraction were filtered using a 1.0/0.45-μm PET double-syringe filter and then purified using Supelclean ™ LC-SAX SPE 57017 column. The column was preactivated with 3 ml of methanol, followed by 2 ml of MiliQ water. The organic acids retained on the column were eluted with 20 mm NaH_2_PO_4_ buffer solution and then filtered through a 0.20-μm filter.

Identification and quantification of sugars in each sample was performed using Acquity UPLC H-Class (Waters, Milford, Massachusetts, USA) equipped with a Waters 410 refractive index (RI) detector at the Parque Tecnológico Aula Dei [PCTAD, currently Fundación de Innovación y Transferencia Agroalimentaria de Aragón (FITA; Zaragoza, Spain). The column chromatography was Flavour Green Ca2+ (8 μm, 8 × 300 mm). The sugars were separated in HiperSolv Chomanorm Water using isocratic method with a flux 0.8 ml/min and a 10-μl injection volume [63]. For acids, the same equipment was used with a photodiode array (PDA) detector, and with an ACQUITY UPLC HSS T3 (1.8 μm, 2.1 × 100 mm) column and an ACQUITY HSS T3 1.8 μM VANGUARD Pre-Col (Waters, Milford, Massachusetts, USA) precolumn. Acid separation was performed using a 20 mm NaH_2_PO_4_ buffer solution with a flux of 0.2 ml/min.

### Statistical analysis

The mean, standard deviation, minimum, and maximum values of all compounds were calculated for each population in both years. In each population, segregation normality was tested for both years according to the Shapiro–wilk test (*P*-value < 0.001). Significant differences in sugar content and soluble solids between annual means were studied for each population. The *T*-student and Man-Whitney *U*-tests were used to perform a mean comparison (*P*-value < 0.05). Correlations between the traits within years in all the analyzed individuals were calculated using the Spearman coefficient (*ρ*; *P*-value < 0.001). Broad-sense heritability (*H^2^*) for SSC, TA, and each sugar and organic acid was estimated from the data of two years using the equation: ${H}^2=\frac{\sigma_g^2}{\sigma_g^2+\frac{\sigma_e^2}{n}}$ where ${\sigma}_g^2$ is the variance of genotype effect, ${\sigma}_e^2$ is the variance of the residual term, and *n* is the number of years. These analyses were performed using the software R v4.1.1 [64].

### QTLs analyses

Sugars, organic acids, SSC, and TA phenotyping data were used for QTL mapping using genotypic data and genetic maps generated previously [65]. QTL mapping was conducted using the FlexQTL™ [66, 67] as described by [27]. This software provides the possibility to study several families simultaneously, increasing the probability of detecting quantitative trait loci (QTL) [67]. For each trait, finite polygenic models were assumed with additive gene effect [68] and Markov Chain Monte Carlo simulations were performed with a minimum of 250 000 interactions to obtain at least 100 effective chain samples with the objective of having at least a sample of 100 samples per simulation [67]. Four simulations were performed, varying the prior number of the QTLs (1 and 3) and the seed number to create independence between iterations in order to verify the consistency of the results. The inference on the number of QTLs was estimated using the natural log of Bayes factors (2lnBF). This parameter was interpreted as positive (2–5), strong (5–10), or decisive (>10) evidence for the presence of QTLs. The QTL positions are based on posterior QTL intensities and the inference on QTL contributions are based on the posterior mean estimates of the QTL effect sizes [66]. QTLs were named according to the standard QTL nomenclature guidelines recommended in the Genome Database for Rosaceae [e.g. *qP-SSC4.2^m^*: where *q* = quantitative trait; *P* = Prunus; SSC = trait (e.g. soluble solid content); 4 = chromosome number; 2 = second chronological QTL reported for this trait on this chromosome; and m = QTL identified in multiple years] [46].

### Haplotype analyses

Haplotype analysis was carried out in the major stable QTL identified for SSC and sugars, and TA and acids, as described by Calle et al. [69]. The haplotypes were obtained from SNP phase estimated by FlexQTL™. SNPs identified in the most significant QTL region were selected, inheritance in the populations was confirmed, and recombinant individuals in these regions were discarded. The mean phenotypic values for SSC, sugars, TA, and malic acid, were calculated for all the population individuals for each QTL haplotype each year. These values were then compared using ANOVA or Kruskal–Wallis (*P*-value < 0.05) with the software R.

## Supplementary Material

Web_Material_uhae310

## Data Availability

The datasets generated in this study are available in the Genome Database for Rosaceae (https://www.rosaceae.org/publication_datasets) number tfGDR1083.
