# Supplementary material for: Genetic and QTL analyses of sugar and acid content in sweet cherry (Prunus avium L.)
Source: Hortic Res. 2024 Nov 6;12(2):uhae310. doi: 10.1093/hr/uhae310 (PMC11818002; doi:10.1093/hr/uhae310)
Supplement: Web_Material_uhae310 [file web_material_uhae310.zip › Supl_Fig.pdf]

(A)

2021 \ 2019	Glucose	Fructose	Sorbitol	Sucrose	SSC
Glucose	**0.16***	0.86***	0.85***	0.73***	0.67***
Fructose	0.74***	**0.04**	0.67***	0.77***	0.47***
Sorbitol	0.73***	0.55***	**0.53****	0.55***	0.77***
Sucrose	0.54***	0.39***	0.23***	**-0.22****	0.37***
SSC	0.70***	0.63***	0.84***	0.14*	**0.41**

(B)

2021 \ 2019	Malic	Quinic	Oxalic	Citric	Shikimic	TA
Malic	**0.33*****	0.32***	0.26***	0.07	0.28***	0.45***
Quinic	0.26***	**0.19****	0.46***	0.15*	0.52***	0.2**
Oxalic	0.41***	0.6***	**0.32*****	0.3***	0.34***	0.29***
Citric	-0.03	0.24***	0.43***	**0.05**	0.07	0.2**
Shikimic	0.15*	0.58***	0.28***	0.02	**0.27*****	0.14*
TA	0.47***	0.03	0.11	-0.13	0.04	**0.29*****

Supplementary Figure 1: Spearman correlation coefficients between sugars and SSC (A), and between organic acids and TA (B), in 2019 (above diagonal, yellow) and in 2021 (below diagonal, blue). The diagonal corresponds to correlation between the two years, calculated for the individuals (n=222) evaluated both years. Significance: p-value < 0.001***; p-value <0.01 **; p-value < 0.05 *.

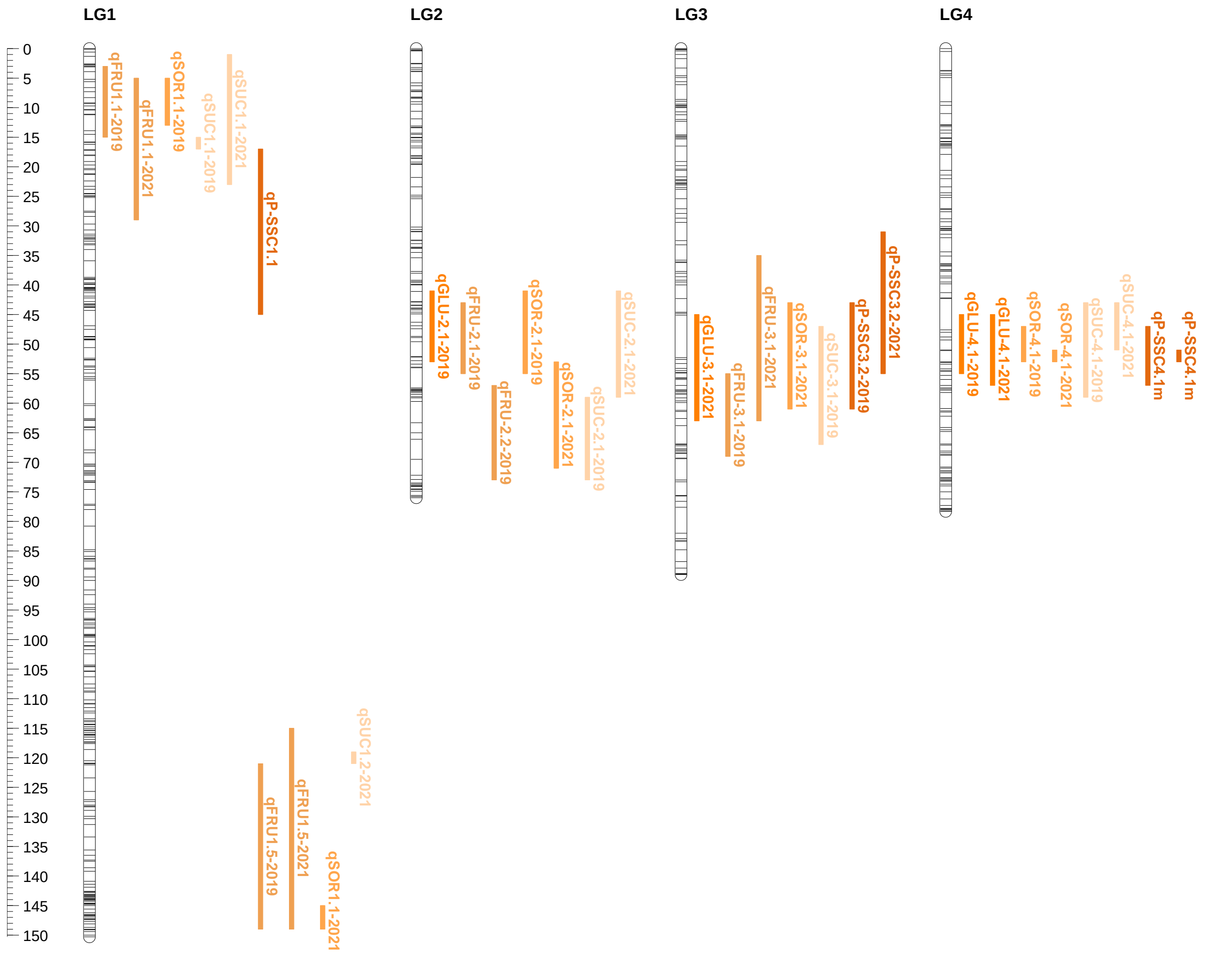

Supplementary Figure 2: Genetic position on (cM) on the consensus linkage map (Calle et al., 2020) of sugar QTLs colocalized in the same regions of the genome in different years. Sugar QTLs for glucose (GLU), fructose (FRU), Sorbitol (SOR) and sucrose (SUC).

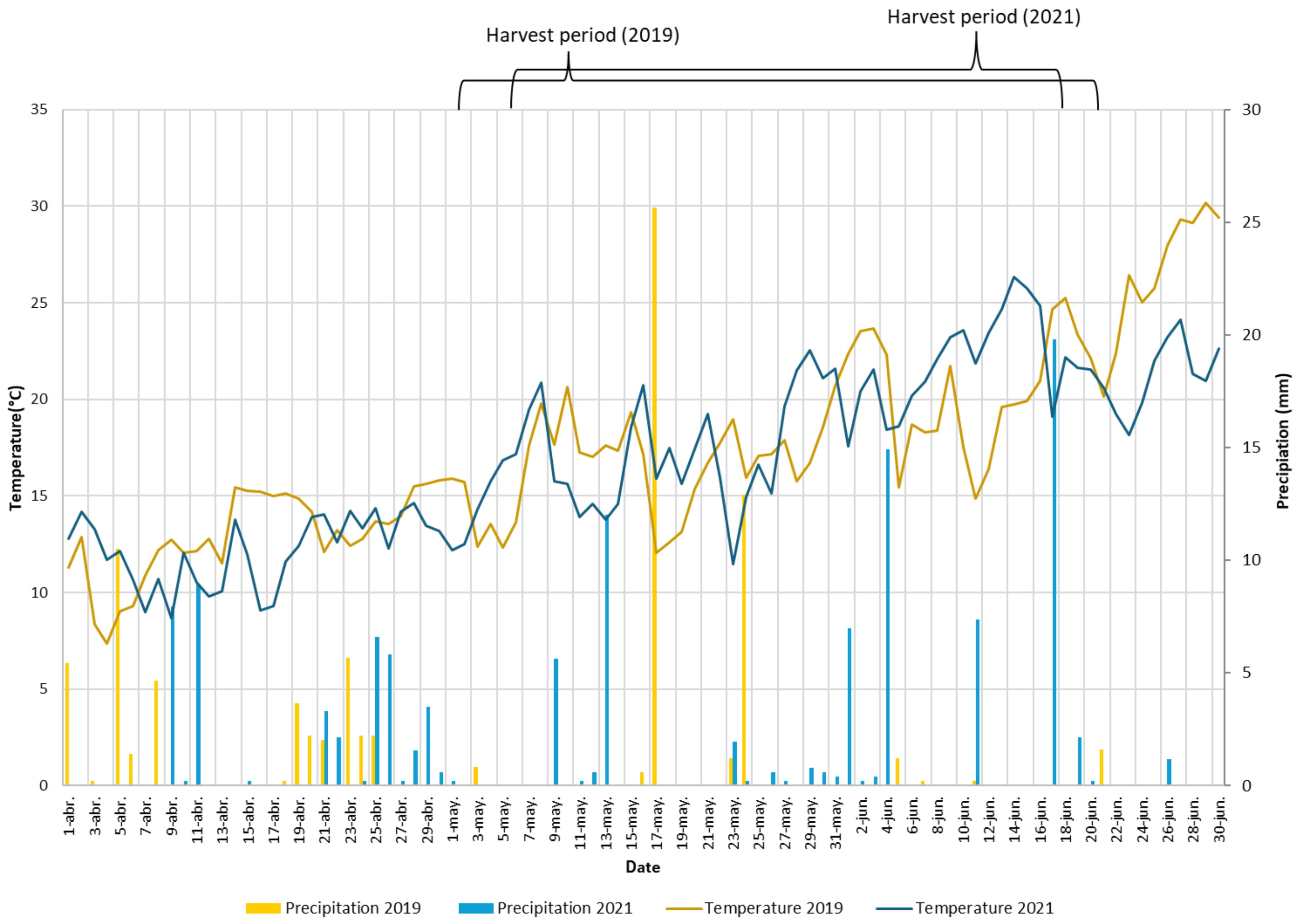

Supplementary Figure 3: Temperature and precipitation data for the two analyzed years. The left axis shows temperature in degrees Celsius (°C), and the right axis represents precipitation in millimeters (mm). Meterological data from SIAR ("Sistema de Información Agroclimática para el Regadío", Ministerio de Agricultura, Pesca y Alimentación, Gobierno de España; https://servicio.mapa.gob.es/websiar)
