# Supplementary material for: Genetic and QTL analyses of sugar and acid content in sweet cherry (Prunus avium L.)
Source: Hortic Res. 2024 Nov 6;12(2):uhae310. doi: 10.1093/hr/uhae310 (PMC11818002; doi:10.1093/hr/uhae310)
Supplement: Web_Material_uhae310 [file web_material_uhae310.zip › Supl_Tables.pdf]

Supplementary Table 1: Phenotypic values of SSC and sugar content in the parental and ancestor cultivars. FW: Fresh weight.

	Glucose (g/100g FW)		Fructose (g/100g FW)		Sorbitol (g/100g FW)		Sucrose (g/100g FW)		SSC (°Brix)	
	**2019**	**2021**	**2019**	**2021**	**2019**	**2021**	**2019**	**2021**	**2019**	**2021**
**Ambrunés**	3.84	3.88	3.68	3.09	1.34	1.57	0.76	0.73	18.5	20
**Bing**	2.28	3.68	1.92	3.03	1.26	1.49	0.65	1.13	22.1	18.6
**Brooks**	4.73	4.72	4.48	3.78	1.77	1.76	1.09	0.98	22.7	23.8
**Burlat**	3.50	2.02	3.15	1.84	0.81	0.43	0.57	0.72	17.4	15.2
**Cristobalina**	3.42	3.53	3.47	3.02	1.19	1.15	0.73	0.78	19.3	18.6
**Lambert**	3.42	3.76	2.34	2.75	1.03	1.28	0.90	1.05	16.5	17.5
**Napoleon**	4.06	3.72	3.69	2.83	1.72	1.59	1.11	0.84	20.8	23
**Rainer**	3.90	3.49	3.70	2.97	1.64	1.27	0.86	1.00	12.9	17.6
**Van**	4.33	4.21	4.04	3.36	1.49	1.81	1.23	0.73	21.6	22.1
**Vic**	4.58	4.61	3.64	3.15	1.75	1.92	1.00	1.19	21.7	22.5

Supplementary Table 2: Phenotype value of SSC and sugar content in each population for years 2019 and 2021. Mean values, standard deviations (SD), value ranges (range), and number of individuals analyzed (N) are shown. FW: Fresh weight.

		Glucose (g/100g FW)		Fructose (g/100g FW)		Sorbitol (g/100g FW)		Sucrose (g/100g FW)		SSC (°Brix)	
**Population**		**2019**	**2021**	**2019**	**2021**	**2019**	**2021**	**2019**	**2021**	**2019**	**2021**
**A×C**	**Mean ± SD**	3.75 ± 1.6	4.12 ± 0.66	3.25 ± 0.88	3.4 ± 0.41	1.67 ± 0.55	1.82 ± 0.53	0.69 ± 0.19	0.89 ± 0.14	20.50 ± 2.35	21.16 ± 2.91
	**Range**	2.39 - 6.99	3.31 - 6.84	2.88 - 6.42	2.45 - 4.13	0.99 - 3.18	0.98 - 3.22	0.39 - 1.27	0.47 - 1.13	16.9 - 26.3	16.7 - 26.8
	**N**	39	38	39	38	39	38	39	38	39	38
**BC2**	**Mean ± SD**	3.71 ± 0.38	3.63 ± 0.35	3.42 ± 0.47	3.39 ± 0.32	1.36 ± 0.46	1.48 ± 0.51	0.88 ± 0.22	0.74 ± 0.22	19.09 ± 2.24	20.18 ± 2.02
	**Range**	3 - 4.48	3.47 - 4.38	2.63 - 4.42	2.74 - 4.13	0.48 - 2.17	0.56 - 2.54	0.52 - 1.2	0.42 - 1.87	14.7 - 23.2	17.3 - 24.3
	**N**	23	28	23	28	23	28	23	28	23	28
**C×C**	**Mean ± SD**	3.66 ± 0.81	3.93 ± 0.82	3.16 ± 0.71	3.66 ± 0.62	1.75 ± 0.48	2.39 ± 0.55	0.66 ± 0.16	0.79 ± 2.57	21.75 ± 1.86	23.10 ± 2.79
	**Range**	1.91 - 7.16	1.85 - 5.74	1.61 - 6.29	1.95 - 4.7	0.85 - 3.52	0.92 - 3.27	0.32 - 1.29	0.37 - 1.15	17.0 - 24.8	18.2 - 30.1
	**N**	34	38	34	38	34	38	34	38	35	37
**L×C**	**Mean ± SD**	4.3 ± 0.79	3.83 ± 0.79	3.33 ± 0.53	2.72 ± 0.45	1.84 ± 0.63	1.34 ± 0.5	0.85 ± 0.15	0.88 ± 0.14	22.91 ± 4.67	18.65 ± 2.66
	**Range**	2.98 - 5.7	2.96 - 5.19	2.75 - 3.94	2.2 - 4.67	1.19 - 2.73	0.8 - 2.35	0.53 - 1.3	0.74 - 1.13	17.2 - 30.8	14.9 - 23.3
	**N**	9	10	9	10	9	10	9	10	9	10
**V×C**	**Mean ± SD**	4.0 ± 0.57	3.61 ± 0.55	3.65 ± 0.4	3.18 ± 0.42	1.84 ± 0.39	1.85 ± 0.47	0.89 ± 0.17	0.62 ± 0.13	20.99 ± 2.41	21.54 ± 2.38
	**Range**	2.34 - 5.34	1.67 - 5.12	2.34 - 4.58	1.82 - 3.78	1.5 - 2.94	0.77 - 2.98	0.46 - 1.26	0.34 - 1.14	16.1 - 27.1	17.2 - 28.9
	**N**	139	149	139	149	139	149	139	149	142	149
**Total**	**Mean ± SD**	3.89 ± 0.7	3.73 ± 0.63	3.48 ± 0.6	3.27 ± 0.48	1.76 ± 0.46	1.79 ± 0.52	0.82 ± 0.2	0.76 ± 0.19	20.91 ±2.52	21.45 ± 2.65
	**Range**	1.91 - 7.16	1.67 - 6.84	1.61 - 6.42	1.82 - 4.67	0.48 - 3.52	0.56 - 3.27	0.32 - 1.3	0.34 - 1.15	14.7-30.8	14.9 - 30.1
	**N**	244	263	244	263	244	263	244	263	248	262

Supplementary Table 3: Phenotypic values of organic acids content and titratable acidity (TA) in parental and ancestor cultivars. FW: Fresh weight.

	Malic acid (mg/100g FW)		Quinic acid (mg/100g FW)		Oxalic acid (mg/100g FW)		Citric acid (mg/100g FW)		Shikimic acid (mg/100g FW)		TA (%)	
	**2019**	**2021**	**2019**	**2021**	**2019**	**2021**	**2019**	**2021**	**2019**	**2021**	**2019**	**2021**
**Ambrunés**	219.08	279.09	47.30	86.07	14.8	23.27	8.4	1.40	2.50	4.21	0.81	0.88
**Bing**	181.58	399.95	34.50	59.03	17.50	17.58	2.5	0.97	1.00	1.14	0.72	1.05
**Brooks**	308.72	126.83	48.90	76.30	17.90	21.92	5.60	0.81	1.40	0.18	0.95	0.97
**Burlat**	247.63	179.32	36.70	50.24	24.10	14.67	6.10	5.41	1.50	1.26	0.90	0.71
**Cristobalina**	193.13	260.76	50.70	83.45	25.30	26.69	4.4	9.00	1.00	1.35	0.77	0.98
**Lambert**	258.52	260.70	28.90	43.45	22.10	15.00	1.30	0.41	1.70	1.32	0.92	0.92
**Napoleon**	371.41	89.02	51.80	37.00	24.50	10.87	5.2	0.62	1.90	0.82	1.15	1.07
**Rainer**	179.95	128.50	31.90	41.44	14.50	9.83	5.00	0.71	2.30	1.11	1.10	0.88
**Van**	433.51	392.04	52.10	71.68	20.00	26.54	2.70	1.18	1.60	2.2	1.16	1.22
**Vic**	324.81	346.34	51.10	109.11	25.70	28.06	6.8	2.59	3.10	2.1	0.96	0.91

Supplementary Table 4: Phenotype value of organic acids content and titratable acidity (TA) in each population for years 2019 and 2021. Mean values, standard deviations (SD), interval range (range), and number of individuals analyzed (N) are shown. FW: Fresh weight.

		Malic (mg/ 100g FW)		Quinic (mg/100g FW)		Oxalic (mg/100g FW)		Citric (mg/100g FW)		Shikimic (mg/100g FW)		TA (mg/100g FW)	
**Population**		**2019**	**2021**	**2019**	**2021**	**2019**	**2021**	**2019**	**2021**	**2019**	**2021**	**2019**	**2021**
**AxC**	**Mean**	183.37±64.55	266.94±90.32	42.225±11.48	92.232±28.95	22.53±7.01	21.775±5.13	7.261±3.65	3.239±1.51	1.601±0.39	1.949±0.51	0.958±0.14	0.987±0.13
	**Range**	36.62-308.63	76.88-461.59	19.41-73.45	53.93-161.31	6.94-42.07	8.5-34.94	2.59-16	0.74-7.38	0.69-2.41	0.8-3.53	0.72-1.3	0.77-1.27
	**N**	38	36	38	36	38	36	38	36	38	34	38	38
**BC2**	**Mean**	249.35±92.94	322.291±56.39	39.805±11.2	80.934±18.3	22.846±5.12	24.28±4.2	5.789±4.4	3.161±1.7	1.275±0.22	1.658±0.37	0.99±0.14	0.98±0.07
	**Range**	36.77-395.14	229.84-474.64	20.7-61.48	53.23-117.32	11.1-32.31	17.43-31.48	0.8-16.19	1.01-7.86	0.86-1.72	0.73-2.49	0.77-1.23	0.88-1.19
	**N**	23	28	23	28	23	28	23	28	23	28	23	28
**CxC**	**Mean**	167.274±70.24	232.562±88.54	55.637±14.36	121.427±28.85	32.752±7.6	32.119±6.53	11.689±5.57	7.518±2.26	1.504±0.39	1.88±0.54	1.011±0.11	0.927±0.11
	**Range**	24.68-321.16	33.26-438.58	27.19-87.47	64.43-165.63	18.98-50.02	19.74-42.97	4.5-24.13	2.8-11.42	0.84-2.32	1.1-3.18	0.75-1.19	0.71-1.23
	**N**	34	36	33	35	33	36	31	35	33	36	35	37
**LxC**	**Mean**	244.157±99.97	292.556±89.9	58.19±30.57	73.469±22.71	25.738±7.56	24.705±9.59	5.626±4.67	2.753±1.07	2.163±1.11	2.179±0.72	1.042±0.22	0.983±0.11
	**Range**	115.45-381.54	80.37-413.45	31.52-107.89	30.46-103.33	15.29-37.64	15.11-43.63	1.05-13.6	0.88-4.24	1.1-3.78	0.77-3.17	0.76-1.4	0.81-1.14
	**N**	9	9	9	9	9	9	9	9	9	9	8	10
**VxC**	**Mean**	222.96±66.18	310.945±99.05	56.405±16.8	73.353±20.9	26.591±6.18	24.301±5.72	7.898±5.01	3.278±1.54	1.93±0.62	1.41±0.49	0.944±0.13	0.978±0.12
	**Range**	53.32-412.45	42.28-575.38	27.24-103.79	26.34-139.65	11.09-45.31	12.35-43.05	0.92-23.01	0.86-8.46	0.82-3.79	0.16-2.91	0.62-1.39	0.71-1.35
	**N**	142	147	142	148	142	148	137	148	141	146	141	149
**Total**	**Mean**	212.391±74.56	294.329±96.4	52.609±17.18	83.414±28.31	26.408±7.09	25.054±6.47	8.001±5.05	3.821±2.21	1.768±0.6	1.604±0.55	0.963±0.14	0.973±0.12
	**Range**	24.68-412.45	33.26-575.38	19.41-107.89	26.34-165.63	6.94-50.02	8.5-43.63	0.8-24.13	0.74-11.42	0.69-3.79	0.16-3.53	0.62-1.4	0.71-1.35
	**N**	246	256	245	256	245	257	238	256	244	253	245	262

Supplementary Table 5: Broad- sense heritability (H^2) of sugars, SSC, organic acids, and TA in each population and in all populations.

Population	Glucose	Fructose	Sorbitol	Sucrose	SSC	Malic	Quinic	Oxalic	Citric	Shikimic	TA
**AxC**	0.24	0.35	0.75	0.18	0.83	0.01	0.59	0.42	<0.01	0.83	0.35
**BC2**	0.57	0.60	0.86	<0.01	0.74	0.62	0.48	0.20	<0.01	0.67	0.21
**CxC**	0.03	0.07	0.48	<0.01	0.46	0.41	0.98	0.60	0.23	0.76	0.70
**LxC**	0.76	0.75	0.93	0.89	0.93	0.83	0.82	0.82	0.50	0.43	0.61
**VxC**	0.37	0.13	0.68	0.10	0.53	0.05	0.37	0.18	<0.01	0.11	0.49
**All**	0.23	0.08	0.72	<0.01	0.62	0.58	0.32	0.49	0.16	0.41	0.43

Supplementary Table 6: Significant sugars and solid soluble content (SSC) QTLs identified each year. Trait, year of detection, linkage group (LG), QTL interval in cM, QTL peak (interval position with largest 2lnBF), maximum 2ln Bayes Factor (Max2lnBF), average of 2lnBF, mean additive effect, percentage of variance explained (PVE) are shown.

Trait	Year	QTL name	LG	Interval (cM)	QTL Peak(cM)	Max2lnBF	Average 2lnBF	Additive effect	PVE (%)
**Glucose (GLU)**	2019	*qP-GLU2.1*	2	41-53	49	4.18	3.04	2.725	3.56
	2019	*qP-GLU4.1*^{m}	4	45-55	51	9.71	7.18	4.082	25.22
	2021	*qP-GLU3.1*	3	45-63	59	8.99	6.64	1.74	8.08
	2021	*qP-GLU4.1*^{m}	4	45-57	53	11.38	6.49	3.38	24.07
	2021	*qP-GLU5.1*	5	59-69	63	3.88	3.27	1.21	< 1
	2021	*qP-GLU6.1*	6	89-95	93	4.45	2.53	1.47	9.35
**Fructose (FRU)**	2019	*qP-FRU1.1*^{m}	1	3-15	11	4.27	3.91	1.95	4.06
	2019	*qP-FRU1.2*	1	51-67	59	5.93	4.97	2.71	12.55
	2019	*qP-FRU1.5*^{m}	1	121-149	141	4.09	2.49	2.14	2.76
	2019	*qP-FRU2.1*	2	43-55	51	4.36	3.76	3.53	6.62
	2019	*qP-FRU2.2*	2	57-73	67	8.86	6.18	3.94	44.74
	2019	*qP-FRU3.1*^{m}	3	55-69	59	5.97	4.57	2.77	8.14
	2021	*qP-FRU1.1*^{m}	1	5-29	21	6.81	3.78	0.96	3.40
	2021	*qP-FRU1.3*	1	83-99	95	7.36	4.38	1.31	3.23
	2021	*qP-FRU1.4*	1	107-111	109	4.57	3.63	1.81	1.66
	2021	*qP-FRU1.5*^{m}	1	115-149	129	5.88	3.96	3.64	1.10
	2021	*qP-FRU3.1*^{m}	3	35-63	45	7.92	5.31	1.15	6.08
	2021	*qP-FRU5.1*	5	53-71	67	8.32	6.09	1.78	14.39
	2021	*qP-FRU6.1*	6	81-97	89	7.42	5.08	1.10	3.53
	2021	*qP-FRU6.2*	6	105-109	109	6.69	5.69	1.15	1.21
**Sorbitol (SOR)**	2019	*qP-SOR1.1*	1	5-13	7	3.88	2.88	0.89	0.56
	2019	*qP-SOR2.1*^{m}	2	41-55	47	9.28	6.17	2.68	28.65
	2019	*qP-SOR4.1*^{m}	4	47-53	51	12.21	8.53	3.42	27.48

Supplementary Table 6: continued

**Sorbitol (SOR)**	2021	*qP-SOR1.2*	1	67-69	67	10.08	6.60	3.85	16.44
	2021	*qP-SOR2.1*[m]	2	53-71	63	6.05	4.67	2.73	12.00
	2021	*qP-SOR3.1*	3	43-61	59	10.24	6.56	1.65	9.53
	2021	*qP-SOR4.1*[m]	4	51-53	53	12.17	11.77	5.32	51.45
	2021	*qP-SOR6.1*	6	13-25	21	4.09	3.30	1.43	2.32
	2021	*qP-SOR6.2*	6	77-97	85	5.36	3.59	0.84	1.05
**Sucrose (SUC)**	2019	*qP-SUC1.1*[m]	1	15-17	16	2.59	2.59	0.36	< 1
	2019	*qP-SUC2.3*	2	59-73	67	4.75	3.75	0.85	5.85
	2019	*qP-SUC3.1*	3	47-67	53	6.23	5.05	0.98	18.51
	2019	*qP-SUC3.4*	3	81-89	87	4.89	3.43	1.05	4.46
	2019	*qP-SUC4.2*[m]	4	43-59	49	6.16	4.44	0.38	4.41
	2019	*qP-SUC5.2*	5	57-61	57	3.66	3.06	1.13	2.55
	2019	*qP-SUC8.1*[m]	8	31-57	57	4.75	2.95	7.12	2.55
	2021	*qP-SUC1.1*[m]	1	1-23	1	5.14	2.72	0.60	1.96
	2021	*qP-SUC1.2*	1	119-121	119	3.26	2.70	0.27	< 1
	2021	*qP-SUC2.1*	2	27-37	27	3.11	2.52	0.7	1.15
	2021	*qP-SUC2.2*	2	41-59	45	5.62	3.10	0.66	3.75
	2021	*qP-SUC4.1*	4	1-33	15	5.52	4.13	0.68	7.00
	2021	*qP-SUC4.2*[m]	4	43-51	45	3.11	2.28	0.19	< 1
	2021	*qP-SUC5.1*	5	3-29	17	5.36	3.22	0.60	5.12
	2021	*qP-SUC6.1*	6	9-43	17	5.84	4.17	0.87	11.89
	2021	*qP-SUC8.1*[m]	8	37-55	55	6.63	3.89	0.94	7.30
**Solid soluble content (SSC)**	2019	*qP-SSC1.1*	1	17-45	33	3.66	2.73	0.61	3.58
	2019	*qP-SSC1.2*	1	65-73	71	4.82	2.88	0.48	< 1
	2019	*qP-SSC2.1*	2	37-51	43	6.89	4.528	1.074	10.53
	2019	*qP-SSC3.2*[m]	3	43-61	45	4.60	2.923	0.37	< 1
	2019	*qP-SSC4.1*[m]	4	47-57	51	10.94	7.052	1.52	32.81
	2019	*qP-SSC7.1*	7	27-71	47	7.40	5.125	0.80	14.45

Supplementary Table 6: continued

**Solid soluble content (SSC)**	2021	*qP-SSC3.1*	3	3-9	5	2.75	2.48	0.42	1.98
	2021	*qP-SSC3.2*^{m}	3	31-55	35	6.59	4.15	0.87	11.91
	2021	*qP-SSC4.1*^{m}	4	51-53	51	11.25	9.55	2.08	38.07
	2021	*qP-SSC6.1*	6	5-19	17	3.05	2.16	0.24	1.33
	2021	*qP-SSC6.2*	6	81-109	105	4.17	2.78	0.34	2.64
	2021	*qP-SSC8.1*	8	51-61	55	4.36	3.41	0.52	5.76

Supplementary Table 7: Organic acids content and titratable acidity (TA) QTLs identified each year. Trait, year of detection, linkage group (LG), QTL interval in cM, QTL peak (interval position with largest 2lnBF), maximum 2ln Bayes Factor (Max2lnBF), average of 2lnBF, mean additive effect, percentage of variance explained (PVE) are shown.

Trait	Year	QTL name	LG	Intervale(cM)	Peak(cM)	Max2lnBF	Average 2lnBF	Additive effect	PVE (%)
**Tiratable Acidity (TA)**	2019	*qP-TA1.1*^{m}	1	7-29	17	6.233	3.821	0.066	5.26
	2019	*qP-TA3.1*	3	77-81	81	2.775	2.031	0.032	<1
	2019	*qP-TA6.1*^{m}	6	87-99	95	6.006	4.331	0.048	<1
	2019	*qP-TA7.1*	7	49-73	67	9.434	5.731	0.089	15.79
	2019	*qP-TA8.1*^{m}	8	29-41	31	3.534	2.659	0.057	<1
	2021	*qP-TA1.1*^{m}	1	13-25	25	3.263	2.560	0.050	<1
	2021	*qP-TA3.2*	3	81-87	83-85	3.774	3.464	0.073	<1
	2021	*qP-TA6.1*^{m}	6	87-109	95	8.987	5.182	0.047	7.14
	2021	*qP-TA8.1*^{m}	8	23-35	27	2.775	2.182	0.041	<1
**Malic (MAL)**	2019	*qP-MAL3.1*	3	1-9	3	6.407	4.507	44.875	3.13
	2019	*qP-MAL3.2*	3	31-55	37	6.994	5.503	55.296	16.32
	2019	*qP-MAL3.3*	3	85-89	87	3.534	2.951	31.401	<1
	2019	*qP-MAL6.1*	6	65-77	69	6.776	5.107	39.176	3.30
	2019	*qP-MAL6.2*^{m}	6	85-99	95	10.049	6.688	37.574	11.11
	2019	*qP-MAL7.2*	7	41-75	67	5.139	3.191	32.709	2.43
	2021	*qP-MAL2.1*	2	37-51	37	4.445	2.588	52.309	1.13
	2021	*qP-MAL6.2*^{m}	6	85-97	95	12.360	6.001	49.837	13.70
	2021	*qP-MAL6.3*	6	105-109	107	4.678	4.343	45.417	0.80
	2021	*qP-MAL7.1*	7	9-15	9	3.989	2.913	50.773	0.90
**Quinic (QUI)**	2019	*qP-QUI1.1*	1	33-39	37	2.132	1.828	8.558	<1
	2019	*qP-QUI1.2*	1	65-75	71	3.774	2.647	8.061	10.00
	2019	*qP-QUI3.1*	3	61-67	63	2.132	1.591	7.361	<1
	2019	*qP-QUI4.1*^{m}	4	45-59	53	9.264	7.399	10.224	16.67
	2019	*qP-QUI7.1*	7	19-51	31	7.591	4.014	7.976	13.33
	2019	*qP-QUI7.2*	7	59-73	61	2.951	1.824	6.535	6.67
	2021	*qP-QUI1.3*	1	119-145	141	4.985	3.986	24.978	22.40
	2021	*qP-QUI4.1*^{m}	4	47-53	51	12.425	5.756	17.377	19.34

Supplementary Table 7: continued

**Oxalic (OXA)**	2019	*qP-OXA1.1*	1	1-29	13	4.678	2.922	3.372	2.23
	2019	*qP-OXA1.2*	1	65-73	67	4.526	3.263	2.772	0.62
	2019	*qP-OXA1.3*^{m}	1	113-151	147	5.615	4.067	5.888	8.26
	2019	*qP-OXA2.1*^{m}	2	63-75	69	3.263	2.940	4.290	1.81
	2019	*qP-OXA4.1*	4	1-37	1	4.603	3.588	4.609	6.35
	2019	*qP-OXA4.2*	4	61-67	61	3.774	2.884	3.620	<1
	2019	*qP-OXA6.2*	6	67-109	75	5.841	3.762	4.469	10.56
	2019	*qP-OXA7.1*	7	39-71	65	4.820	3.163	3.205	2.66
	2021	*qP-OXA1.3*^{m}	1	95-149	137	5.797	4.214	5.298	21.13
	2021	*qP-OXA2.1*^{m}	2	41-73	69-71	4.274	2.805	4.012	3.64
	2021	*qP-OXA3.1*	3	1-31	25	4.678	2.760	5.008	4.88
	2021	*qP-OXA3.2*	3	69-83	77	2.132	2.010	2.452	0.40
	2021	*qP-OXA6.1*	6	1-13	11	4.183	2.322	4.338	1.81
	2021	*qP-OXA8.1*	8	43-49	45	3.774	3.456	5.344	1.58
**Citric (CITR)**	2019	*qP-CITR2.1*	2	31-47	45	6.986	4.233	2.090	6.83
	2019	*qP-CITR7.1*	7	53-75	63	4.421	3.446	2.239	6.06
	2021	*qP-CITR1.1*	1	67-69	67	7.988	7.424	3.539	10.56
	2021	*qP-CITR2.2*	2	63-75	71	7.422	5.065	3.019	12.45
	2021	*qP-CITR4.1*	4	43-59	53	11.508	6.398	1.730	11.97
	2021	*qP-CITR8.1*	8	41-53	47	6.994	5.634	3.160	15.02
**Shikimic (SHIK)**	2019	*qP-SHIK1.1*	1	65-69	67	14.104	9.658	0.274	<1
	2019	*qP-SHIK4.1*^{m}	4	49-53	51	12.583	8.559	0.691	<1
	2019	*qP-SHIK5.1*	5	53-71	61	6.693	5.236	0.171	<1
	2019	*qP-SHIK7.1*	7	15-47	23	9.405	4.197	0.225	<1
	2021	*qP-SHIK1.1*	1	57-61	57	3.589	3.130	0.154	0.67
	2021	*qP-SHIK2.1*	2	59-73	65	5.341	4.415	0.264	7.02
	2021	*qP-SHIK4.1*^{m}	4	49-53	51	11.268	7.199	0.363	23.077
	2021	*qP-SHIK7.2*	7	55-59	57	2.870	2.538	0.155	2.341

Supplementary Table 8. Parental haplotypes in the 50-54 cM region of LG4 QTLs (Calle & Wünsch, 2020) for SSC (*qP-SSC4.1^m*), glucose (*qP-GLU4.1^m*), sorbitol (*qP-SOR4.1^m*), sucrose (*qP-SUC4.2^m*), quinic (*qP-QUI4.1^m*) and shikimic (*qP-SHIK4.1^m*) acids.

		**SNPs** (Genetic position in cM)					
Parental Cultivars	Parental Haplotypes	ss490559054 (50.965)	ss490552906 (51.086)	ss490552928 (53.030)	ss490552931 (53.127)	ss490548726 (53.410)	ss490552936 (53.412)
'Ambrunes'	*H4-a*	B	A	B	A	A	A
	H4-a	B	A	B	A	A	A
'BC8'	*H4-d*	B	A	A	A	B	B
	H4-c	B	B	A	B	A	A
'Bing'	*H4-b*	A	A	B	A	A	A
	H4-b	A	A	B	A	A	A
'Brooks'	*H4-b*	A	A	B	A	A	A
	H4-d	B	A	A	A	B	B
'Burlat'	*H4-c*	B	B	A	B	A	A
	H4-d	B	A	A	A	B	B
'Cristobalina'	*H4-c*	B	B	A	B	A	A
	H4-a	B	A	B	A	A	A
'Lambert'	*H4-b*	A	A	B	A	A	A
	H4-b	A	A	B	A	A	A
'Napoleon'	*H4-a*	B	A	B	A	A	A
	H4-b	A	A	B	A	A	A
'Rainier'	*H4-b*	A	A	B	A	A	A
	H4-b	A	A	B	A	A	A
'Van'	*H4-b*	A	A	B	A	A	A
	H4-a	B	A	B	A	A	A
'Vic'	*H4-b*	A	A	B	A	A	A
	H4-a	B	A	B	A	A	A

Supplementary Table 9: Parental haplotypes in 95-96cM region of LG6 QTLs for TA (*qP-TA6.1* ^{m}) and malic acid (*qP-MAL6.2* ^{m})

		**SNPs** (Genetic position in cM)			
Parental Cultivars	Parental Haplotypes	ss490556190 95.717	ss490559031 95.759	ss490559034 95.76	ss490556194 95.955
'Ambrunes'	*H6-c*	B	B	A	A
	H6-b	A	A	B	A
'BC8'	*H6-c*	B	B	A	A
	H6-c	B	B	A	A
'Bing'	*H6-a*	A	A	B	B
	H6-b	A	A	B	A
'Brooks'	*H6-b*	A	A	B	A
	H6-c	B	B	A	A
'Burlat'	*H6-c*	B	B	A	A
	H6-c	B	B	A	A
'Cristobalina'	*H6-b*	A	A	B	A
	H6-c	B	B	A	A
'Lambert'	*H6-b*	A	A	B	A
	H6-a	A	A	B	B
'Napoleon'	*H6-a*	A	A	B	B
	H6-b	A	A	B	A
'Rainier'	*H6-a*	A	A	B	B
	H6-b	A	A	B	A
'Van'	*H6-b*	A	A	B	A
	H6-b	A	A	B	A
'Vic'	*H6-a*	A	A	B	B
	H6-b	A	A	B	A

Supplementary Table 10: Mean phenotypic values, variance and mean comparison of haplotypes (*H4-a,-b,-c,-d*) in LG4 QTLs for SSC (*qP-SSC4.1^m*), glucose (*qP-GLU4.1^m*), sorbitol (*qP-SOR4.1^m*), quinic (*qP-QUI4.1^m*) and shikimic (*qP-SHIK4.1^m*) acids, calculated for all individuals in the populations studied. Means comparison was calculated between haplotypes within each year. Letters (a, b, c, d) indicate significant differences (p-value<0.05) between means.

		**Haplotypes**			
	Year	*H4-a*	*H4-b*	*H4-c*	*H4-d*
SSC (°Brix)	2019	21.50±2.49a	21.25±3.02a	19.90±2.14b	19.95±1.96b
	2021	22.17±2.47a	21.45±2.84b	20.34±2.52bc	20.95±2.36c
Glucose (g/100FW)	2019	4.03±0.73a	4.04±0.63a	3.64±0.65b	3.85±0.37a
	2021	3.89±0.64a	3.73±0.58b	3.56±0.62c	3.74±0.5ab
Sorbitol (g/100FW)	2019	1.89±0.46a	1.85±0.44a	1.53±0.41b	1.54±0.36b
	2021	1.95±0.47a	1.81±0.51b	1.52±0.46c	1.69±0.55b
Quinic (mg/100FW)	2019	56.19±17.16a	55.19±18.63a	47.95±15.4b	42.51±12.12b
	2021	88.64±29.56a	75.45±23.88b	78.88±27.92b	87.54±19.66a
Shikimic (mg/100FW)	2019	1.99±0.64a	1.96±0.66a	1.45±0.35b	1.31±0.24b
	2021	1.73±0.59a	1.53±0.57b	1.47±0.43b	1.73±0.46a

Supplementary Table 11: Mean phenotypic values, variance and mean comparison of haplotypes (*H6-a,-b,-c*) in LG6 QTLs for TA (*qP-TA6.1* [m]) and malic acid (*qP-MAL6.2* [m]), calculated for all individuals in the populations studied. Means comparison was calculated between haplotypes within each year. Letters (a, b) indicate significant differences (p-value<0.05) between means.

		**Haplotypes**		
	Year	*H6-a*	*H6-b*	*H6-c*
TA	2019	0.92±0.14b	0.96±0.13ab	0.98±0.14a
	2021	0.95±0.12b	0.97±0.12b	0.99±0.12a
Malic acid (mg/100 g FW)	2019	207.8±64.43a	212.2±75.29a	221.63±81.91a
	2021	280.68±99.94b	284.16±101.35b	308.47±90.08a
